# Ruxolitinib as a Novel Therapeutic Option for Poor Prognosis T-LBL Pediatric Patients

**DOI:** 10.3390/cancers13153724

**Published:** 2021-07-24

**Authors:** Giulia Veltri, Chiara Silvestri, Ilaria Gallingani, Max Sandei, Sara Vencato, Federica Lovisa, Giuliana Cortese, Marta Pillon, Elisa Carraro, Silvia Bresolin, Alessandra Biffi, Giuseppe Basso, Benedetta Accordi, Lara Mussolin, Valentina Serafin

**Affiliations:** 1 Maternal and Child Health Department, Division of Pediatric Hematology, Oncology and Stem Cell Transplant, University of Padova, 35128 Padova, Italy; giulia.veltri@phd.unipd.it (G.V.); silvestrikiara@gmail.com (C.S.); ilaria.gallingani@gmail.com (I.G.); federica.lovisa@unipd.it (F.L.); silvia.bresolin@unipd.it (S.B.); alessandra.biffi@unipd.it (A.B.); giuseppe.basso@unipd.it (G.B.); benedetta.accordi@unipd.it (B.A.); lara.mussolin@unipd.it (L.M.); 2 Fondazione Città della Speranza Istituto di Ricerca Pediatrica, Oncohematology, Stem Cell Transplant and Gene Therapy Research Area, 35122 Padova, Italy; max.sandei90@gmail.com (M.S.); sara.vencato94@gmail.com (S.V.); 3 Department of Statistical Sciences, University of Padova, 35121 Padova, Italy; gcortese@stat.unipd.it; 4 Clinic of Pediatric Oncohematology, University of Padova, 35128 Padova, Italy; marta.pillon@unipd.it (M.P.); elisa.carraro87@gmail.com (E.C.)

**Keywords:** T-LBL, ruxolitinib, Jak2, resistance

## Abstract

**Simple Summary:**

Current treatment protocols for pediatric patients with T-Lymphoblastic lymphoma (T-LBL) allow the achievement of a complete remission in around 85% of T-LBL pediatric patients; however the overall survival rate of second-line treatments for patients with progressive disease or relapse is around 14%. Thus, the major issues to be addressed are the identification of a valuable predictor marker to foresee the disease risk and new therapeutic targets to improve relapsed/resistant patients’ outcome. We identified JAK2 Y1007-1008 as a potential prognosis marker as well as a therapeutic target for patients with progressive disease or relapse and suggest that its inhibition by ruxolitinib, a JAK1/2 FDA approved inhibitor, could represent a novel therapeutic approach to overcome therapy resistance and meliorate the outcome of pediatric T-LBL patients.

**Abstract:**

Lymphoblastic lymphoma (LBL) is the second most common type of non-Hodgkin lymphoma in childhood, mainly of T cell origin (T-LBL). Although current treatment protocols allow a complete remission in 85% of cases, the second-line treatment overall survival for patients with progressive or relapsed disease is around 14%, making this the major issue to be confronted. Thus, we performed a Reverse Phase Protein Array study in a cohort of 22 T-LBL patients to find reliable disease risk marker(s) and new therapeutic targets to improve pediatric T-LBL patients’ outcome. Interestingly, we pinpointed JAK2 Y1007-1008 as a potential prognosis marker as well as a therapeutic target in poor prognosis patients. Hence, the hyperactivation of the JAK1/2-STAT6 pathway characterizes these latter patients. Moreover, we functionally demonstrated that STAT6 hyperactivation contributes to therapy resistance by binding the glucocorticoid receptor, thus inhibiting its transcriptional activity. This was further confirmed by specific STAT6 gene silencing followed by dexamethasone treatment. Finally, JAK1/2-STAT6 pathway inhibition by ruxolitinib, an FDA approved drug, in cell line models and in one T-LBL primary sample led to cell proliferation reduction and increased apoptosis. Globally, our results identify a new potential prognostic marker and suggest a novel therapeutic approach to overcome therapy resistance in pediatric T-LBL patients.

## 1. Introduction

Lymphoblastic lymphoma (LBL) is the second most common type of non-Hodgkin lymphoma (NHL) in childhood and adolescence, accounting for 25–35% of all cases. The majority of LBLs, 70–80%, originate from T cell precursors and express T cell markers corresponding to T cell intrathymic differentiation stages, while 20–25% arise from B lymphoblasts [1,2]. Current treatment protocols for T-LBL use a wide variety of different chemotherapeutic agents in combination, such as glucocorticoids, asparaginase, daunorubicin, cyclophosphamide, methotrexate and vincristine to eradicate the bulk of lymphoma cells [3]. Although this approach allows for the achievement of an event-free survival (EFS) rate from 75% to 85% [4,5], the overall survival rate of second-line treatment for patients with progressive or relapsed disease is around 14% [6], making the prognosis of these patients extremely poor. Thus, the major issues to be addressed are the identification of a valuable predictor marker to foresee the disease risk in pediatric T-LBL and new therapeutic targets to improve relapsed/resistant patients’ outcome.

To this end, we analyzed by Reverse Phase Protein Arrays (RPPA) [7,8] the phosphoproteomic profile in a cohort of T-LBL patients at diagnosis and of T-LBL and T-ALL cell lines. Intriguingly, we pointed out that JAK2 Y1007-1008 hyperphosphorylation could be a potential new biomarker of therapy resistance and/or relapse at diagnosis as well as a new therapeutic target in T-LBL patients. Moreover, we observed a general hyperactivation of the JAK1/2-STAT6 pathway in patients with disease progression and/or who relapsed (poor prognosis) compared to patients who achieved complete remission (good prognosis). To date, aberrant regulation of the JAK1/2-STAT6 pathway has been identified in different pathological conditions [9] and activated STAT6 has been found in patient samples isolated from Hodgkin lymphomas [10,11], cutaneous T cell lymphomas [12], adult T cell leukemia and B-lymphomas [13,14]. In this article we functionally validated JAK2 Y1007-1008 as a new therapeutic target to reverse therapy resistance by in vitro treatment with ruxolitinib, an already FDA approved JAK1/2 inhibitor [15,16] that is currently also being evaluated for the treatment of B-ALL (NCT02723994) [17], alone or in combination with dexamethasone, in cell lines and in one T-LBL pediatric primary sample. Moreover, our findings also revealed that in these cells STAT6 controls the transcriptional activity of the glucocorticoid receptor (GR) through the binding of GR itself and the inhibition of its activity, which contributes to increasing glucocorticoid (GC) resistance. Taken together, our results suggest a novel mechanism of resistance to therapy and that treatment of poor prognosis T-LBL patients with ruxolitinib could be a valuable therapeutic option to improve the outcome of these patients.

## 2. Results

### 2.1. RPPA Analysis Reveals JAK2 Y1007-1008 as a Potential Biomarker of Poor Prognosis for T-LBL Patients at Diagnosis

**Figure 1:**
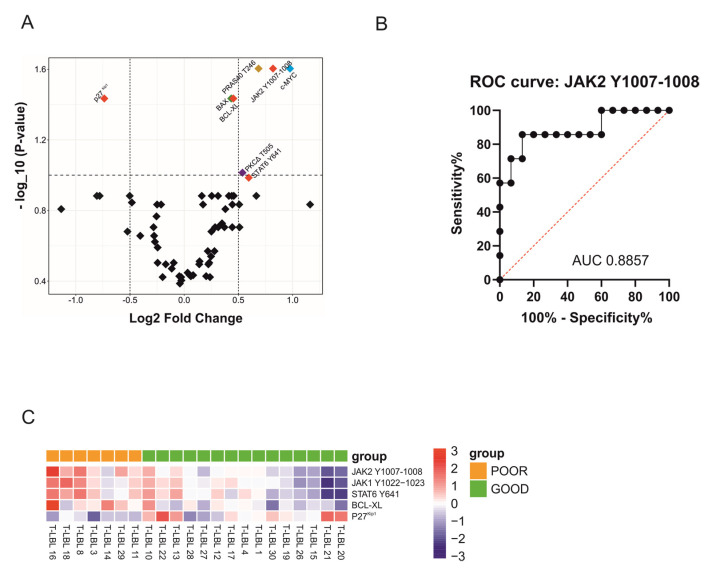
JAK1/2-STAT6 pathway in T-LBL pediatric patients. (**A**) Volcano plot of all proteins evaluated by RPPA. Log2 Fold Change, given for each protein, is computed as the difference in the means of log base 2 between good vs. poor prognosis. A Log2 Fold Change equal to 0 indicates no difference, higher than 0 indicates that the mean in patients with good prognosis is higher than the mean for poor prognosis. Statistical significance for each protein is reported in the vertical axes (as -log base 10 of the q-values). Q-values ≤ 0.1 are equivalent to points above (or on) the horizontal dashed line. In red, proteins belonging to the JAK1/2-STAT6 pathway (JAK2 Y1007-1008, STAT6, BCL-XL, p27^kip1^), in orange PRAS40 T246, in blue c-MYC, in green BAX and in violet PKCΔ T505. (**B**) Receiver operating characteristic (ROC) analysis of the signal intensities obtained by RPPA raw data for JAK2 Y1007-1008 expression in T-LBL patients. (**C**) Heatmap showing the activation status of the proteins belonging to the JAK1/2-STAT6 signaling pathway in good vs. poor prognosis T-LBL patients. RPPA raw data were log2 transformed and each intensity value was plotted and scaled by row.

We employed RPPA analysis on a cohort of 22 pediatric T-LBL patients (Table S1) to explore differential protein expression/activation among the 64 proteins belonging to the most deregulated pathways in cancer and that could be correlated with patients’ prognosis (Table S2). Despite the unsupervised hierarchical cluster analysis not revealing an evident cluster between poor and good prognosis patients (Figure S1A), we identified a group of eight proteins with a significant test result (Figure 1A, colored dots and Table S1), that have been further studied as valuable predictor markers and/or a therapeutic target for patients’ prognosis. Remarkably, we found that four out of these eight proteins belong to the JAK2-STAT6 pathway (Figure 1A, red dots). Specifically, JAK2 Y1007-1008 (q = 0.025, local FDR = 0.045), BCL-XL (q = 0.036, local FDR = 0.078) and STAT6 Y641 (q = 0.103, local FDR = 0.148) expression was higher, whereas p27^Kip1^ expression was lower (q = 0.037, local FDR = 0.079) in patients with poor prognosis (Figure S1B). We then wondered whether any of these four significant proteins could represent a potential biomarker of poor prognosis already at diagnosis for these patients. Thus, we performed area under the ROC curve (AUC) analysis that revealed that JAK2 Y1007-1008 (Figure 1B), compared to STAT6 Y641, BCL-XL and p27^kip1^ (Figure S2A), achieved the best AUC value (AUC = 0.88; *p* = 0.0043, CI 95% 71,81 to 100%), with a high sensitivity (71.43%) and specificity (93.33%) selected on the higher likelihood ratio value it could represent a putative prognostic biomarker to improve T-LBL patients’ stratification at diagnosis, although a validation cohort should be further analyzed for JAK2 Y1007-1008 hyperphosphorylation.

### 2.2. Hyperactivation of JAK1/2-STAT6 Pathway Characterizes T-LBL Pediatric Patients with Poor Prognosis

To verify whether this pathway was globally different between the T-LBL patients that achieve remission and the ones with worse prognosis, we performed a global test [18] analysis considering all the 22 patients and all the analyzed proteins belonging to the JAK1/2-STAT6 pathway (JAK1 Y1022-23, JAK2 Y1007-1008, STAT6 Y641, p27^Kip1^ and BCL-XL). This latter analysis revealed that the JAK1/2-STAT6 pathways is hyperactivated in T-LBL patients with poor prognosis compared to patients with good prognosis (*p* = 0.00158). The heatmap shows JAK1/2-STAT6 pathway activation in good vs. poor prognosis T-LBL pediatric patients (Figure 1C). Finally, this result is in good agreement with the correlation matrix in the poor prognosis T-LBL subgroup that reveals a strong positive correlation between JAK2 Y1007-1008 and JAK1 Y1022-23, STAT6 Y641 and BCL-XL protein phosphorylation/expression (Pearson ρ = 0.72, ρ = 0.82 and ρ = 0.50, respectively), and a negative one with p27^kip1^ expression (ρ = 2.08 e−^003^) (Figure S2B). Of note, considering that p27^kip1^ can be regulated both by STAT6 [19] and miR-221, as we previously demonstrated in pediatric T-LBL [20] patients, we investigated miR-221 expression in good and poor prognosis T-LBL patients, but we did not observe any difference between the two groups (Figure S2C), thus suggesting that p27^Kip1^ down-regulation is likely driven by STAT6 as previously reported also by other groups [19]. We also evaluated whether the JAK1/2-STAT6 activation was associated with any of the clinical features listed in Table S1, and we observed that higher levels of JAK1/2-STAT6 pathway significantly stratify patients based on the PFS (*p* = 0.039) (Figure S2D) and that there is a positive association with the outcome and the high level of JAK1/2-STAT6 activation (Fisher test; *p* < 0.1, Table S4). Altogether these results prompted us to further investigate the role of the JAK1/2-STAT6 pathway in patients with a poor prognosis.

### 2.3. JAK1/2-STAT6 Pathway Activation by IL-4 Treatment Influences Dexamethasone Response via STAT6

**Figure 2:**
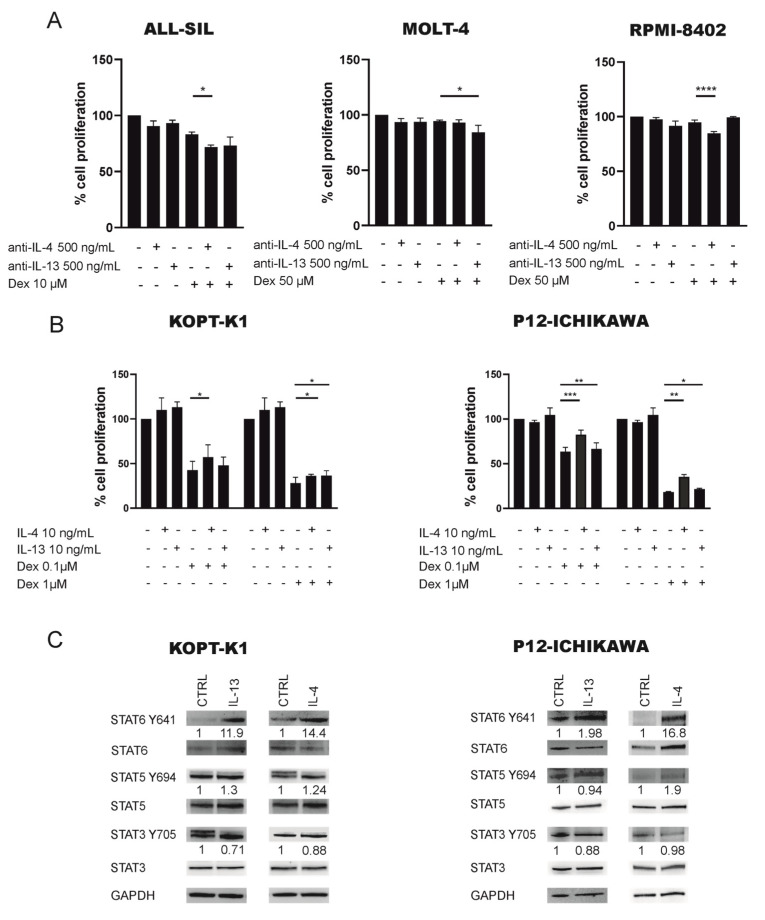
Blockade of IL-4/IL-13 pathway increases the response to dexamethasone by STAT6 activation and GR binding. (**A**) The anti-IL-4 or anti IL-13 neutralizing antibody increases GC-induced cell death in GC resistant cell lines ALL-SIL, MOLT-4 and RPMI-8402. Cells were treated with 500 ng/mL of blocking antibody for 16 h prior to dexamethasone treatment (10 µM for ALL-SIL, 50 µM for MOLT-4 and RPMI-8402 for 48 h), and cell proliferation was evaluated by MTT assay, at least in triplicate. Cell proliferation of control DMSO treated cells was set to 100%. Results are presented as means ± SEM. (**B**) Cell proliferation measured by MTT assay of KOPT-K1 and P12-ICHIKAWA cells treated or not (CTRL) for 16 h with IL-4 or IL-13 (10 ng/mL) followed by dexamethasone (1–0.1 µM) for 48 h (*n* = 3). Cell proliferation of control DMSO treated cells was set to 100%. Results are presented as means ± SEM (*n* = 3) (* *p* < 0.05; ** *p* < 0.01; *** *p* < 0.001; **** *p* < 0.0001. paired t test). (**C**) WB analysis of STAT6 Y641 increase phosphorylation level after IL-4 or IL-13 (10 ng/mL) stimulation of KOPT-K1 and P12-ICHIKAWA cell lines. Of note, STAT5 Y694 and STAT3 Y705 phosphorylation is not increased as well as the respective total form. Intensity values of every protein are normalized on GAPDH first. Following the values of phosphorylated STATs are normalized on the respective total form.

Considering the absence of available patient-derived xenograft (PDX) models and the rareness of fresh materials from T-LBL patients, we identified a suitable in vitro model that could best mimic T-LBL patients with worse prognosis. From RPPA data we performed a hierarchical cluster analysis considering the JAK1/2-STAT6 pathway in T-LBL cases based on good and poor prognosis and 13 commercially available leukemia cell lines (Figure S3A). Of note, ALL-SIL, MOLT-4 and RPMI-8402 cell lines showed a JAK1/2-STAT6 activation profile close to patients with worse prognosis as well as high levels of JAK2 Y1007-1008 compared to good prognosis patients and to the other leukemia cell lines. Thus, to evaluate the potential role of the JAK1/2-STAT6 pathway in contributing to therapy resistance, we next tested in vitro the response of these three selected cell lines to the conventional chemotherapeutics frequently used in T-LBL therapy, namely dexamethasone (dex), cytarabine (AraC), vincristine (Vcr), daunorubicin (dauno), and L-asparaginase (L-Asp) (Sigma-Aldrich, St. Louis, MO, USA) for 48 h. We considered as resistant cell lines with a GI_50_ value ≥ 1 μM; thus, all the three cell lines turned out to be resistant to dexamethasone (Figure S3B). Therefore, we assessed the role of JAK1/2-STAT6 pathway in supporting GC resistance. It is well known that JAK1/2-STAT6 is specifically activated in response to IL-4 and/or IL-13 cytokines binding their cognate receptors on the cell surface membrane. Indeed, both IL-4 and IL-13 cytokines can bind to the IL4 receptor (IL4R) and trigger the signal transduction pathway leading to STAT6 activation [21]. We thus treated these three GC resistant cell lines with either the anti-IL-4 and or anti-IL-13 neutralizing antibody alone or in combination with dexamethasone (Figure 2A). IL-4 blockade in ALL-SIL and RPMI-8402 sensitizes cells to the action of dexamethasone, thus supporting the role of this pathway in sustaining dexamethasone resistance, as previously reported also by our group in prednisone poor responder (PPR) pediatric T-ALL patients [22]. Differently, IL-4 inhibition did not affect MOLT-4 resistance to dexamethasone, while IL-13 blockade sensitizes MOLT-4, but not ALL-SIL and RPMI-8402 cell lines, to the action of dexamethasone, thus suggesting that the activation of the pathway is complementary mediated by both cytokines.

To further investigate the contribution of IL-4 and IL-13 to glucocorticoid response, we selected P12-ICHIKAWA and KOPT-K1 GC sensitive cell lines (Figure S3B) with a JAK1/2-STAT6 activation profile comparable to good prognosis patients (Figure S3A), and we treated these cell lines with IL-4 or IL-13 alone or in combination with dexamethasone (Figure 2B). As expected, MTT tests showed a reduction in dexamethasone response in both IL-4 and IL-13 pretreated cell lines in comparison with controls, suggesting that higher levels of these two cytokines contribute to a diminished response to glucocorticoids. We thus evaluated by Western blotting (WB) the phosphorylation of key components of the IL-4 pathway following IL-4 or IL-13 treatment. Of note, we observed an increased phosphorylation of STAT6 Y641 and not of other STATs, such as STAT5 Y694 and STAT3 Y705 (Figure 2C and Figure S6), similarly to what was observed in T-LBL patients from RPPA analysis where only STAT6 Y641, and no other STATs, were significantly different between good and poor prognosis T-LBL patients (Figure 1A). Overall, these results suggest that GC response can be influenced by IL4 pathway activation and specifically by STAT6. Thus, to verify this latter hypothesis we further investigated the link between STAT6 and GR activity.

### 2.4. STAT6 Mediates GC Resistance by Binding GR and Inhibiting Its Transcriptional Activity

**Figure 3:**
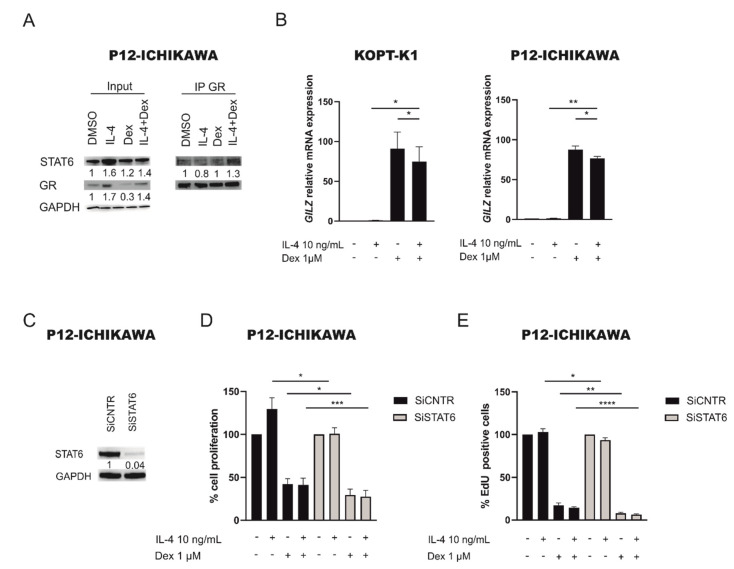
STAT6 binds GR and inhibits its transcriptional activity. (**A**) Co-immunoprecipitation of GR and STAT6 protein in P12-ICHIKAWA cells treated with DMSO, IL-4 (10 ng/mL) or dexamethasone (1 µM) alone, or the combination of IL-4 and dexamethasone. (**B**) GR transcriptional activity measured by *GILZ* mRNA levels in P12-ICHIKAWA and KOPT-K1 cells DMSO treated, stimulated with IL-4 (10 ng/mL ON), dexamethasone (1 µM for 6 h) or the combination of IL-4 and dexamethasone. Results are presented as means ± SEM (*n* = 3). (**C**) WB analysis of STAT6 protein expression in P12-ICHIKAWA SiCNTR and SiSTAT6 after 48 h silencing. (**D**) Cell proliferation determined by MTT test in P12-ICHIKAWA siCNTR (black bars) and in SiSTAT6 (grey bars) followed by treatment with IL-4 (10 ng/mL) or dexamethasone (1 μM) alone or in combination. Cell proliferation of control DMSO treated cells was set to 100%. (**E**) Percentage of EdU positive cells in P12-ICHIKAWA siCNTR (black bars) and in SiSTAT6 (grey bars) followed by treatment with IL-4 (10 ng/mL) or dexamethasone (1μM) alone or in combination. Results are presented as means ± SEM (at least *n* = 3 for all the experiments) (* *p* < 0.05; ** *p* < 0.01; *** *p* < 0.001; **** *p* < 0.0001. paired t test).

It has been previously described that in normal T cell lymphocytes the transcriptional activity of the GR can be inhibited by STAT6 [23] binding. We thus wondered whether this mechanism could contribute to dexamethasone resistance also in our model. As first, to verify whether STAT6 and GR physically interact, we performed an immunoprecipitation for GR protein in P12-ICHIKAWA cell line (Figure 3A, Figures S4 and S6) after treatment with IL-4 and dexamethasone alone or in combination, and we evaluated the binding of STAT6. Interestingly, we observed that after stimulation with IL-4 or treatment with dexamethasone there is no increase in STAT6 binding to GR. Conversely, in the combined treatment with IL-4 and dexamethasone there is more STAT6 protein bound to GR compared to untreated or dexamethasone only treated cells, thus supporting our idea that in the presence of IL-4 and dexamethasone, STAT6 can bind to GR and inhibits its activity. To verify this latter hypothesis, we evaluated GR transcriptional activity by measuring *GILZ* mRNA expression, a well-known GR target gene [24], in P12-ICHIKAWA and KOPT-K1 cells after IL-4 or dexamethasone treatment alone or in combination. Interestingly, we observed that after dexamethasone treatment *GILZ* mRNA levels increased both in P12-ICHIKAWA and KOPT-K1 cell lines, whereas *GILZ* expression significantly decreased if cells were pretreated with IL-4 before dexamethasone treatment (Figure 3B), thus also explaining the decreased response to dexamethasone treatment in IL-4 or IL-13 pretreated GC sensitive cell lines (Figure 2B). To finally demonstrate that this mechanism is STAT6 mediated, we performed specific STAT6 gene silencing in P12-ICHIKAWA cells (Figure 3C and Figure S6) followed by 48 h treatment with dexamethasone combined or not with a 16 h IL-4 pre-treatment. Of note, both P12-ICHIKAWA SiSTAT6 cells treated with dexamethasone alone or pre-treated with IL-4 followed by 48 h of dexamethasone treatment showed an increased response to GC compared to SiCNTR cells cultured at the same experimental conditions (Figure 3D,E, third and fourth gray bar). We did not observe an increased GC resistance in SiCNTR cells pre-stimulated with IL-4 followed by dexamethasone treatment, different to what observed in Figure 2B, probably due to the stress induced by the electroporation process.

**Figure 4:**
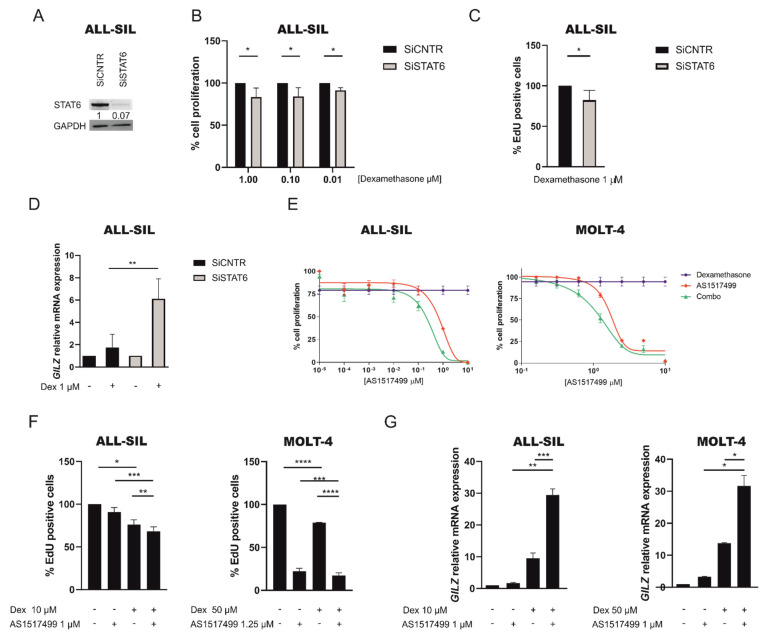
Inhibition of STAT6 sensitizes GC resistant cells to the action of dexamethasone. (**A**) WB analysis of STAT6 protein expression in ALL-SIL cells control (SiCNTR) and silenced (SiSTAT6) after 48 h of silencing. (**B**) ALL-SIL cell proliferation was determined by MTT test after specific gene silencing of STAT6 and treatment with dexamethasone at scalar doses for 48 h. Cell proliferation of control cells was set to 100%. Results are presented as means ± SEM (at least *n* = 3 for all the experiments). (**C**) Percentage of EdU positive cells in ALL-SIL SiCNTR and SiSTAT6 cells after treatment with dexamethasone at 1 μM for 48 h. Cell proliferation of control cells was set to 100%. Results are presented as means ± SEM. (**D**) GR transcriptional activity measured by *GILZ* mRNA levels in ALL-SIL cells control and STAT6 silenced after treatment with DMSO or dexamethasone (1 μM) for 6 h. Results are presented as means ± SEM (*n* = 3). (**E**) Cell proliferation, determined by MTT test, in ALL-SIL and MOLT-4 cells after 48 h of treatment with scalar doses of AS1517499 and fixed dose of dexamethasone, 10 µM or 50 µM, respectively, for ALL-SIL and MOLT-4 alone or in combination. Results are presented as means ± SEM (*n* = 3). (**F**) Percentage of EdU positive cells in ALL-SIL and MOLT-4 cell lines after treatment with DMSO, AS1517499 (1 µM) and dexamethasone at (10 µM or 50 µM) alone or in combination for 48 h. Cell proliferation of control cells was set to 100%. Results are presented as means ± SEM (**G**) GR transcriptional activity measured by *GILZ* mRNA levels in ALL-SIL and MOLT-4 treated with DMSO, AS1517499 (1 µM) and dexamethasone (10 µM or 50 µM) alone or in combination over-night. Results are presented as means ± SEM, (*n* = 3 at least for all experiments). (* *p* < 0.05; ** *p* < 0.01; *** *p* < 0.001; **** *p* < 0.0001. paired t test).

To further demonstrate the pivotal role of STAT6 in contributing to GC resistance, we performed both STAT6 specific gene silencing and pharmacological inhibition with the AS1517499 inhibitor, which selectively inhibits STAT6 [25], in GC resistant cell lines alone or in combination with dexamethasone. As expected, in ALL-SIL cell line in which the expression of STAT6 was knocked down (Figure 4A and Figure S6), we observed a significant cell proliferation decrease after 48 h of dexamethasone treatment at 1–0.01 μM compared to control measured both by MTT assay and EdU staining (Figure 4B,C). Moreover, the knock-down of STAT6 in ALL-SIL followed by dexamethasone treatment led to a significantly augmented GR activity, measured by *GILZ* expression, compared to control (Figure 4D). Similarly, when we treated GC resistant ALL-SIL and MOLT-4 cell lines with the AS1517499 STAT6 inhibitor, alone or in combination with dexamethasone, we observed that cell proliferation, measured by MTT assay and EdU staining, was significantly reduced by the combined treatments in both cell lines (Figure 4E,F). Conversely, the GR activity, as measured by *GILZ* expression, was significantly increased in the combined treatment compared to dexamethasone or AS1517499 alone (Figure 4G). The ability of AS1517499 to inhibit STAT6 Y641 phosphorylation was confirmed by WB (Figures S5A and S6) as well as its synergistic activity with dexamethasone, demonstrated by CI values always <1 (Table S5A). Overall, these results clearly show that STAT6 hyperactivation markedly contributes to therapy resistance by binding the glucocorticoid receptor and inhibiting its transcriptional activity.

### 2.5. Inhibition of JAK1/2 by Ruxolitinib Sensitize GC Resistant Cells to Dexamethasone

**Figure 5:**
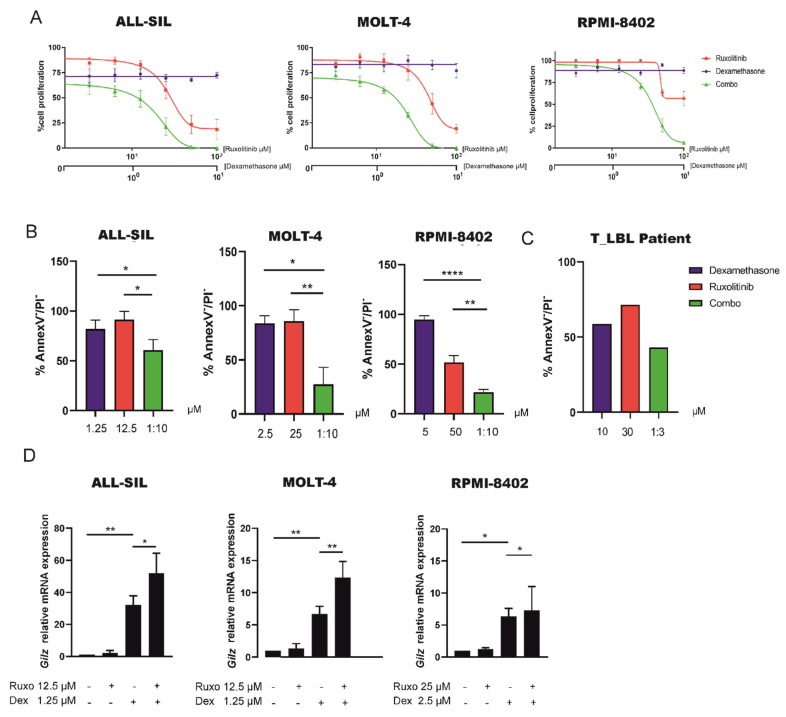
Pharmacological inhibition of JAK1/2 kinases decreases cell proliferation and increases dexamethasone induced cell death in GC resistant leukemia cells. (**A**) ALL-SIL, MOLT-4, and RPMI-8402 cells treated with dexamethasone and ruxolitinib alone or in combination. All the three cell lines were treated at the molar ratio (ruxolitinib: dex) of 10:1 and cell proliferation was determined by MTT test after 72 h of treatment (*n* = 3 at least for all experiments). Results are presented as means ± SEM. (**B**) Cell mortality was determined by flow cytometry and annexin V/PI staining after 72 h of treatment. The percentage of dead cells was established after normalizing cells on DMSO-treated cells. (Paired t test; *n* = 3 for all experiments). Results are presented as means ± SEM. Dexamethasone and ruxolitinib concentrations used in these experiments were selected based on MTT test results. (**C**) Primary cells isolated from one T-LBL patients were treated with dexamethasone (10 μM) and ruxolitinib (30 μM) alone or in combination for 48 h. Cell mortality was determined by flow cytometry and annexin V/PI staining. The percentage of dead cells was established after normalizing cells on DMSO treated cells. (**D**) GR transcriptional activity measured by *GILZ* mRNA levels in ALL-SIL, MOLT-4 and RPMI-8402 cells treated with DMSO, dexamethasone (ALL-SIL and MOLT-4 1.25 μM; RPMI-8402 2.5 μM) or ruxolitinib (ALL-SIL and MOLT-4 12.5 μM; RPMI-8402 25 μM) alone or in combination. Results are presented as means ± SEM (* *p* < 0.05; ** *p* < 0.01; **** *p* < 0.0001. paired t test).

**Figure 6:**
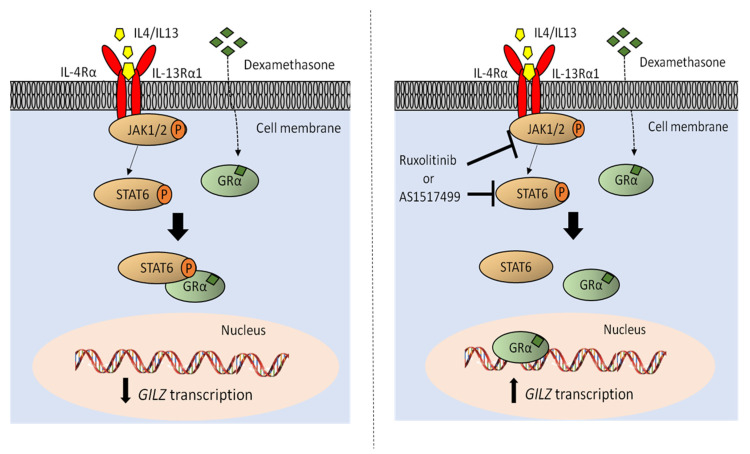
Schematic representation of the JAK1/2-STAT6 pathway and its interaction with GR protein. On the left, the kinases JAK1/2, once activated by the binding of IL-4 or IL-13 to their receptors, can phosphorylate STAT6 in Y641, which in turn sequestrates GR that cannot transcribe its pro-apoptotic genes making these cells more resistant to dexamethasone treatment. On the right the inhibition of JAK1/2 or STAT6 Y641 phosphorylation by ruxolitinib or AS1517499, respectively, allows the GR to bind to the DNA, thus promoting the transcription of its target genes, such as *GILZ*, making these cells more sensitive to dexamethasone treatment.

Considering that there are no other therapeutic options for those patients displaying resistance to therapy or relapse, we evaluated whether ruxolitinib, which specifically targets JAK1/2 and as a consequence the entire downstream pathway including STAT6, could represent a valid therapeutic option for these patients. To this aim, we treated the three cell lines (ALL-SIL, MOLT-4 and RPMI-8402) with a JAK1/2-STAT6 activation profile comparable to T-LBL pediatric patients with worse prognosis, with ruxolitinib alone or in combination with dexamethasone. Intriguingly, cell proliferation was strongly reduced with a synergistic effect (Table S5B) by combined treatments in all the three cell lines (Figure 5A), even if the effect size of drug combinations varied depending on the cell line. Moreover, the ability of ruxolitinib to sensitize cells to dexamethasone was confirmed by annexin V/PI staining in all the three cell lines and, more importantly, in primary cells from one T-LBL pediatric patient (Figure 5B,C). These results strongly suggest that the JAK1/2-STAT6 pathway plays a key role in sustaining GC resistance, and that its activity can be targeted to sensitize these cells to GC treatment. The specificity of ruxolitinib in targeting JAK1/2 pathway was evaluated by WB in all the three cell lines and we observed a general downregulation of the JAK1/2-STAT6 pathway after treatment with ruxolitinib compared to control (Figures S5B and S6). Finally, we also verified whether the combined treatment with ruxolitinib and dexamethasone could increase the GR transcriptional activity. As expected, we observed an increase in *GILZ* mRNA expression in the combined treatment compared to dexamethasone alone in all the three tested cell lines (Figure 5D). These results are in good agreement to what was previously observed after STAT6 inhibition, either by specific gene silencing or treatment with AS1517499, and dexamethasone treatment in ALL-SIL and MOLT4 cell lines (Figure 4D,G). Taken together, these results clearly demonstrate that the inhibition of JAK1/2 kinase activity sensitizes GC resistant cells to dexamethasone by increasing GR transcriptional activity (Figure 6), thus pointing out the potential use of ruxolitinib as an additional new therapeutic strategy for those T-LBL patients that are refractory to therapy already at diagnosis.

## 3. Discussion

Current treatment protocols for T-LBL pediatric patients allow the achievement of EFS rates of approximately 85% [4,5]. However, the overall survival rate of patients with progressive disease or relapse treated with second-line therapies is about 14% [6], making this the most pressing patient population to be addressed. Thus, valuable prognostic markers capable of an early identification of the T-LBL patients at risk of bad/severe prognosis, and new specific drug targets to overcome drug resistance, possibly avoiding high-dose ineffective overtreatments, are highly needed for this patient population.

As of today, the stage of the disease is the only parameter used to stratify T-LBL patients at diagnosis [26]. Recent studies have demonstrated that the presence of minimal disseminated disease in patients’ blood without leukemic bone marrow disease may also predict unfavorable EFS in univariate analysis [27,28]. In addition, activating NOTCH1/FBXW7 mutations were reported to be associated with a favorable prognosis [29], and the mutational status of these two genes will be used for risk group stratification in the forthcoming international protocol. However, we recently demonstrated that NOTCH1 activation could also be present in wild-type cases [30], so a convincing prognostic model for T-LBL has not yet been defined.

To this aim, similar to what other groups performed on DLBCL patients [31], we performed an RPPA analysis comparing protein activation/expression in a pediatric T-LBL patient cohort at diagnosis. We here show that among the most significant proteins evaluated, the hyperphosphorylation of JAK2 Y1007-1008 could represent a new prognostic marker at diagnosis for T-LBL patients with poor prognosis that could benefit from ruxolitinib treatment since diagnosis, although a confirmation of this indication will require an independent validation cohort study by a gold standard technique, such as phospho-flow, which is already used routinely in the diagnostic procedure, to finally propose this biomarker in the new clinical protocol.

To date, several efforts have also been made to identify new therapeutic targets for T-LBL patients. Recently, De Smedt and colleagues [32,33] demonstrated that in a subgroup of T-ALL and T-LBL pediatric patients who were poor responders to dexamethasone treatment, leukemia cells are characterized by the expression of the oncogenic kinase PIM1 via JAK1/2 and STAT5 Y694 phosphorylation and that the inhibition of PIM1 makes these cells sensitive to GC. However, the increased phosphorylation of PIM1 via JAK1/2 and STAT5 Y694 is detectable only after interleukin 7 in vitro stimulation. Differently, in our T-LBL pediatric patient cohort we identified the hyperactivation of the JAK1/2-STAT6 pathway already at basal level in poor prognosis T-LBL patients at diagnosis, thus suggesting a self-autonomous activation loop in this subgroup of patients. Starting from this evidence, we demonstrated that the blockade of the IL-4 pathway in GC resistant cells or its activation in GC sensitive ones leads either to a decrease or an increased GC response, respectively, as also previously demonstrated by our group in T-ALL PPR patients [22]. This evidence is supported by a STAT6 Y641 increased phosphorylation and a GR transcriptional activity reduction that can be explained by the ability of STAT6 to bind the GR, thus inhibiting its transcriptional activity, as previously described in normal T cells [23]. Nevertheless, as per our knowledge, we here provide a first demonstration that this mechanism works also in an oncological context where the increased GC resistance is associated with a STAT6 mediated, decreased GR transcriptional activity. Indeed, this mechanism can be abrogated when we specifically inhibit or knock-down the expression of STAT6 in GC resistant cell lines. This evidence clearly demonstrates from one side the relevance of STAT6 in sustaining leukemia cell proliferation, which is in good agreement also with the role of STAT6 in other types of cancer, such as colon and breast cancer [34] and, more generally, the contribution of the JAK1/2-STAT6 pathway to GC resistance. This novel mechanism can help describe the poor response to treatment of this T-LBL patient subgroup, also in agreement with previous studies on HTLV-1-induced adult T cell leukemia (ATL) [35,36] in which IL-13 and IL-4 were demonstrated to sustain proliferative and antiapoptotic functions that lead to leukemogenesis, and in patients with severe asthma in which IL-4 pathway activation is associated with an increased glucocorticoid insensitiveness [37].

Finally, given the importance of the JAK1/2-STAT6 pathway in sustaining therapy resistance, we clearly demonstrated the importance of targeting T-LBL patients characterized by the hyperactivation of this signaling pathway with ruxolitinib in combination with dexamethasone. Remarkably, inhibition of the JAK1/2-STAT6 pathway induced by ruxolitinib synergized with dexamethasone and reversed GC resistance in all treated cell lines and, more importantly, in primary cells derived from the only available T-LBL pediatric patient. This novel therapeutic approach is also supported by other evidence, mainly proved in T-ALL cases with the finding that early T-cell precursor (ETP) ALL patients with JAK/STAT pathway activation, independently from the presence of JAK/STAT mutations [38], are sensitive to ruxolitinib [39], and that the treatment of IL7R mutant T-ALL cell line or PDX samples with ruxolitinib in combination with dexamethasone has a synergistic effect [40]. Moreover, ruxolitinib showed a potential efficacy also in ATL [41] patients and in patients with glucocorticoid refractory acute versus graft disease (aGVHD) [42,43]. Finally, treatment with ruxolitinib has recently been employed also for COVID-19 patients with hyperinflammation, and a multicenter phase-II clinical trial has been initiated (NCT04338958) [44].

## 4. Material and Methods

### 4.1. Patients

Tumor specimens collected at diagnosis from 22 pediatric T-LBL patients were available in this study for phosphoproteomic analysis. Moreover, one newly diagnosed T-LBL patient pleural effusion sample was used for in vitro treatment with ruxolitinib and dexamethasone. The diagnosis of T-LBL was established from clinical, histological, and immunohistochemistry findings. Tumor biopsies were classified according to WHO guidelines [45]. In all cases, the histological diagnosis was centrally reviewed. Samples were collected at the Pediatric Oncohematology Laboratory (University of Padua, Padua, Italy) between 1999 and 2015. Patients were enrolled in NHL-BFM-type treatment protocols (AIEOP LNH-97 [46] or EuroLB-02 [47]); 18 were males and 4 were females with a median age at diagnosis of 10.1 years (range 2–18 years). All of them were diagnosed with disease at stage III–IV according to the St Jude’s classification [48]. The median follow-up of patients was 5.15 years (range 0.6–12 years). Fifteen patients achieved complete remission after conventional treatment, and seven displayed a worse prognosis characterized by either progression of the disease (*n* = 3), resistance to therapy (*n* = 1) or relapse (*n* = 3). All patients were screened for NOTCH1 and FBWX7 mutations: 60% of good prognosis cases presented mutations in NOTCH1 alone and 6.6% both in NOTCH1 and FBXW7 genes, whereas 42.8% of poor prognosis patients presented mutations in NOTCH1 alone and 14.2% both in NOTCH1 and FBXW7 (Table S1). T-LBL patients’ samples were obtained after informed consent following the tenets of the Declaration of Helsinki. The study was approved by the ethical committee board of the Padova Academic Hospital and the Italian Association of Pediatric Onco-Hematology (AIEOP).

### 4.2. Statistics

Nonparametric two-sample Wilcoxon tests (Mann–Whitney tests) were applied to find differentially activated or expressed proteins between T-LBL patients with poor and good prognosis. Multiplicity corrections were applied following two well-known methods: Storey’s false discovery rate (qvalue) and Storey’s local false discovery rate (local FDR) [49]. The activation status of the JAK1/2-STAT6 pathway was evaluated through a locally most powerful test (global test) [18]. The analyses were performed with the statistical software R 4.0.2 (www.r-project.org, accessed on 23 July 2021). Proteins were considered significantly differentially expressed/activated following two criteria: a q value ≤ 0.1; an FDR and local FDR ≤ 0.15. Pearson’s correlation coefficient was used to determine the relationship between the variables. The RPPA raw intensity values of the biomarker were assumed to come from a continuous quantitative variable, and a nonparametric analysis was performed to estimate the ROC (receiver operating characteristic) curve and the area under the curve (AUC). The Wilson/Brown method was used for the confidence interval estimation. The best cut-off value was selected based on the higher positive likelihood ratio value. Heat maps and clustering analysis to compare the activation and expression levels of the JAK1/2-STAT6 pathway between good and poor prognosis T-LBL patients and between cell line models and T-LBL patients were generated with R software. Fisher test was used to calculate the correlations between JAK1/2-STAT6 RPPA phosphorylation intensity levels and patient clinical and biologic characteristics and clinical outcome.

All in vitro experiments on cell lines were performed at least three times and data are presented as mean ± SEM. Statistical analyses were performed using Prism v.8.4. Cell proliferation difference between cells treated with ruxolitinib and dexamethasone alone or combined was established using paired *t*-test. To determine the synergistic, additive, or agonistic effects of the drug combination from MTT experiments, the Bliss Independence model [50] for combination index (CI) calculation was used. Synergy, additivity, and antagonism were defined by CI < 1, CI = 1 and CI > 1, respectively.

### 4.3. Cell Lines

Human T-LBL cell line SUP-T1 and human T-ALL cell lines RPMI-8402, MOLT-4, ALL-SIL, KOPT-K1, P12-ICHIKAWA, CCRF-CEM, TALL-1, DND-41, MOLT-3, JURKAT, PEER and LOUCY were purchase from DMSZ (Braunschweig, Germany). Cells were cultured in RPMI 1640 (Gibco, Waltham, MA, USA) with 10%–20% Fetal Bovine Serum (FBS) (Gibco), glutamine (2 mM/L; Gibco), penicillin (100 U/mL; Gibco) and streptomycin (100 mg/mL; Gibco) and were maintained at 37 °C in a humidified atmosphere with 5% CO_2_. All cell lines were periodically tested for mycoplasma infection.

### 4.4. In Vitro Treatments

ALL-SIL, MOLT-4 and RPMI-8402 cell lines were pre-treated with anti-IL-4 (#11964; Cell Signaling Technologies, Danvers, MA, USA) or anti-IL-13 human neutralizing antibody (Invivogen, San Diego, CA, USA) 500 ng/mL overnight and then treated with DMSO or dexamethasone (Sigma-Aldrich, St. Louis, MO, USA). Cell proliferation was assessed by (4,5-dimethylthiazol-2-yl) -2,5-diphenyltetrazolium bromide (MTT) test after 72 h. KOPT-K1 and P12-ICHIKAWA cell lines were pre-treated with IL-4 (10 ng/mL, CellS GFH9) or IL-13 (10 ng/mL, CellS GFH85) overnight followed by DMSO or dexamethasone (1–0.1 µM) treatment. Cell proliferation was assessed by MTT after 72 h. ALL-SIL and MOLT-4 were treated with the STAT6 specific inhibitor, AS1517499 alone or in combination with dexamethasone for 48 h. Control cells were treated with DMSO only. Cell proliferation was assessed by MTT. ALL-SIL, MOLT-4 and RPMI-8402 were also treated with ruxolitinib alone or in combination with dexamethasone for 72 h. Control cells were treated with DMSO only. Cell proliferation and viability were assessed by MTT and apoptotic assay (Annexin V/PI), respectively. Primary cells were treated with ruxolitinib and dexamethasone alone or in combination for 48 h and cell viability was evaluated by apoptotic tests (Annexin V/PI). Primary cells were cultured in minimum essential medium α (MEM α; Gibco) with 10% FBS, 10% human serum (Gibco), penicillin (100 U/mL; Gibco), human IL-7 (10 ng/mL; Peprotech, London, UK), human SCF (50 ng/mL; Peprotech), human FLT3- ligand (20 ng/mL; Peprotech), and insulin (20 nM; Sigma-Aldrich). After thawing, the cell viability of primary cells used for Annexin V/PI test, after in vitro treatment with dexamethasone and ruxolitinib, was evaluated by Trypan blue cell count and only samples with a viability >70% were considered.

ALL-SIL SiRNA CNTR and SiRNA STAT6 cells were treated for 48 h with dexamethasone after 24 h from silencing. P12-ICHIKAWA SiRNA CNTR and SiRNA STAT6 cells were stimulated with IL-4 for 16 h after 24 h from silencing and then treated with dexamethasone for 48 h. We could not use the GC sensitive cell line KOPT-K1 in this experiment due to the high mortality of the cells after electroporation.

### 4.5. RPPA

RPPA was performed as previously described [7,8]. Briefly, patient samples and cell lines lysed in an appropriate lysis buffer with protease and phosphatase inhibitor were printed in 4-point dilution curves in quadruplicate on nitrocellulose-coated glass slides (ONCYTE^®^ Nitrocellulose Film Slides, Grace BioLabs, Bend, OR, USA) with the 2470 Arrayer (AushonBioSystems, Billerica, MA, USA). Slides were stained with 64 primary antibodies (Table S2), previously validated by Western blot (WB) for single band specificity, on an automated slide stainer (DakoAutostainer Plus, Dako-Cytomation, Santa Clara, CA, USA). The full list of studied proteins is reported in Table S3. Signal amplification stage was performed through the Amplification System Kit and then signal was revealed using diaminobenzadine/hydrogen peroxide (DAB) as a chromogensubstrate for 5 min (Dako-Cytomation). TIF images of antibody and total protein were analyzed using the Microvigene software (VigeneTech Inc, Carlisle, MA, USA) to extract numeric intensity protein values from the array images.

### 4.6. Western Blotting

Cell pellets were lysed with RIPA buffer (Sigma-Aldrich) following the manufacturer’s instruction. SDS–polyacrylamide gel electrophoresis was performed using 4–20% Criterion TGX Stain Free Protein Gel (Bio-Rad, Hercules, CA), and proteins were transferred to poly-vinylidene difluoride (PVDF) Immobilion-*p* membrane (Merck-Millipore, Billerica, MA, USA) using wet/tank Bio-Rad blotting system. Membranes were blocked in I-block 2% (Invitrogen, Waltham, MA, USA), incubated overnight at 4 °C with primary antibodies and 1 h with the HRP- conjugated secondary antibody (PerkinElmer, Waltham, MA, USA). Bands were detected using Invitrogen™ iBright™ FL1500 Imaging System. The following primary antibodies were used: JAK1 Y1022/1023 (Cell Signaling Technologies, #3331), JAK2 Y1007 (MBS128333, MyBioSource, San Diego, CA, USA), STAT6 Y641 (Millipore, 06-937), STAT3 Y705 (Cell Signaling Technologies, #9145), STAT5 Y694 (Cell Signaling Technologies, #9351), STAT6 (#11290, Becton Dickinson, Franklin Lakes, NJ, USA), STAT3 (79D7) (Cell Signaling Technologies, #4904), STAT5 (Becton Dickinson #611834), GAPDH (GTX 8627408, Genetex, Irvine, CA, USA).

### 4.7. RNA Extraction and Quantitative Real Time-PCR

Total RNA was extracted with RNeasy Mini Kit (Qiagen, Hilden, Germany) following the manufacturer’s instruction. SuperScript^®^ II Reverse Transcriptase (Thermo Fisher Scientific, Waltham, MA, USA) was used for RT-PCR. Real-time PCR was performed using QPCR Platinum Syber Mix (Thermo Fisher Scientific). Relative expression of human *GILZ* gene (fwd: 5′-GAAGGAGCAGATCCGAGAGC-3′, rev:5′-GGCTCAGACAGGACTGGAAC-3′) was calculated following the 2^-ΔΔct^ method, normalizing to the expression of human *GUS* (fwd:5′-GAAAATATGTGGTTGGAGAGCTCATT-3′, rev: 5′-CGGAGTGAAGATCCCCTTTTTA-3′) and to the average ratio of control cells which was arbitrarily defined as 1.

### 4.8. QRT-PCR for miRNA Detection

Total RNA (10 ng) was reverse transcribed by using the TaqMan^®^ MicroRNA Reverse Transcription kit, following the manufacturer’s instructions. Real time PCR was performed in triplicate using TaqMan™ Gene Expression Assay for RNU6B (ID 001093) and miR-221 (ID 000524), with TaqMan™ Fast Advanced Master Mix (Thermo Fisher Scientific).

### 4.9. Co-Immunoprecipitation Assay

P12-ICHIKAWA cells were pre-treated with IL-4 (GFH9, CellS) 10 ng/mL overnight and treated or not with dexamethasone 1 µM for 6 h. Cells were lysed using RIPA buffer. Protein lysate were first incubated with Dynabeads Protein G (Thermo Fisher Scientific) for 30 min at 4 °C. The pre-cleared lysate was then incubated overnight at 4 °C with anti-GR antibody (CS 12041, Cell Signaling Technologies) or goat anti-rabbit IgG-HRP (sc-2004, Santa Cruz Biotechnology, Dallas, TX, USA) as control. Protein–antibody complexes were isolated incubating the lysate with Dynabeads Protein G for 2 h at 4 °C. Immune complexes were washed one time with RIPA buffer and one time with Law salt buffer, before being analyzed by SDS-PAGE using anti-STAT6 (BD 611290, Beckton Dickinson) or anti-GR.

### 4.10. STAT6 Specific Gene Silencing

In P12-ICHIKAWA and ALL-SIL cell lines small-interfering (si)RNA (siSTAT6, Thermo Fisher Scientific) was designed to selectively silence human STAT6. Negative control siRNA (scramble RNA, Thermo Fisher Scientific) was used as control in each experiment. Cell transfection was performed using the Nucleofector systems (Amaxa Biosystems, Lonza Sales Ltd., Basel, Switzerland) according to the manufacturer’s instructions. After 48 h the evaluation of STAT6 silencing was performed through WB analysis.

### 4.11. (3-(4,5-Dimethylthiazol-2-yl)-2,5-diphenyl Tetrazolium Bromide) MTT Assay

Cell proliferation of cell lines was assessed by MTT assay. Equal concentration of cells was plated in a 96-well plate in triplicate and cells were incubated with 10 µL MTT (Sigma-Aldrich) for 4 h. Absorbance was measured at 560 nm using Victor3 TM 1420 Multilabel Counter (PerkinElmer). DMSO treated cells’ viability was set to 100%. The Growth Inhibition 50 (GI_50_, compound concentration required to inhibit cell proliferation by 50%) was calculated by plotting the data as a logarithmic function of (x) when viability was 50%.

### 4.12. EdU Staining

Cell proliferation after STAT6 silencing followed by dexamethasone treatment was assessed after 48 h by EdU staining. Cells were plated in 24-well plate and were incubated with 50µM EdU (BCK-EdU488, Baseclick, Munich, Germany) for 2 h. Following this, cells were fixed with 3.7% formaldehyde and permeabilized with Triton 0.5% X-100. Afterwards, cells were incubated for 30 min at room temperature with a reaction cocktail prepared following manufacturer’s instructions. Samples were analyzed by flow cytometric analysis (Cytomics FC500, Beckman Coulter, Brea, CA, USA).

### 4.13. Apoptotic Assay

Cell viability after ruxolitinib and dexamethasone treatments alone or in combination was assessed after 72 h for cell lines and 48 h for primary cells by Annexin V-FLUOS staining kit (Roche, Basel, Switzerland), following manufacturer’s instructions. Samples were analyzed by flow cytometric analysis (FC500, Cytomics, South San Francisco, CA, USA).

## 5. Conclusions

Our study reveals that JAK2 Y1007-1008 hyperphosphorylation could be a putative marker of poor prognosis already at diagnosis in T-LBL pediatric patients, as well as a potential therapeutic target for these latter patients. Thus, the addition of ruxolitinib, a known FDA approved drug that targets JAK1/2, to conventional chemotherapy could represent a new valuable therapeutic option to improve the outcome of T-LBL patients.

## Data Availability

The data presented in this study are available on request from the corresponding author.

## Supplementary Materials

The following are available online at https://www.mdpi.com/article/10.3390/cancers13153724/s1. Table S1: Patients’ characteristics, Table S2: List of *q*-values and local FDR, Table S3: List of primary antibodies selected for RPPA staining, Table S4: Correlations between JAK1/2-STAT6 RPPA phosphorylation intensity levels and patient clinical and biologic characteristics and clinical outcome, Table S5: Combination index (CI) in ALL-SIL, MOLT-4 and RPMI-8402 GC resistant cell lines (MTT test), Figure S1: RPPA results, Figure S2: Evaluation of potential biomarkers of prognosis at diagnosis in T-LBL patients and protein correlation matrix, Figure S3: Selection of the in vitro model and sensitivity of selected cell lines to the conventional chemotherapeutic compounds, Figure S4: Uncropped images of co-immunoprecipitation of GR and STAT6 protein in P12-ICHIKAWA cells treated with DMSO, IL-4, dexamethasone or the combination of IL-4 and dexamethasone, Figure S5: JAK1/2-STAT6 levels after AS1517499 or ruxolitinib treatment in ALL-SIL, MOLT-4 and RPMI-8402, Figure S6. uncropped original western blot.
