# Supplementary material for: Ruxolitinib as a Novel Therapeutic Option for Poor Prognosis T-LBL Pediatric Patients
Source: Cancers (Basel). 2021 Jul 24;13(15):3724. doi: 10.3390/cancers13153724 (PMC8345121; doi:10.3390/cancers13153724)
Supplement: Supplementary file 1 [file cancers-13-03724-s001.zip › cancers-1273726-supplementary.pdf]

cancers MDPI

Supplementary Material: Ruxolitinib as a Novel Therapeutic Option for Poor Prognosis T-LBL Pediatric Patients

Giulia Veltri, Chiara Silvestri, Ilaria Gallingani, Max Sandei, Sara Vencato, Federica Lovisa, Giuliana Cortese, Marta Pillon, Elisa Carraro, Silvia Bresolin, Alessandra Biffi, Giuseppe Basso, Benedetta Accordi, Lara Mussolin and Valentina Serafin

Table S1. Patients characteristics.

Code	Sex	Age	Stage	PTEN mut	FBXW7 mut	NOTCH1 mut	Event	% Blasts	Immunophenotype	Complete remission	Protocol
**T-LBL 1**	M	9,68	IV	0	0	1	0	0,4	T-LBL	induction	LNH-97
**T-LBL 3**	M	4,21	III	0	0	0	1	na	T-LBL	not achieved	EuroLB02
**T-LBL 4**	M	15,36	III	0	0	0	0	na	T-LBL	consolidation	LNH-97
**T-LBL 8**	M	10,56	III	0	0	1	1	na	T-LBL	not achieved	LNH-97
**T-LBL 10**	F	16,56	III	0	0	1	0	na	T-LBL	consolidation	LNH-97
**T-LBL 11**	M	14,57	III	0	0	0	1	na	preB	re-induction	LNH-97
**T-LBL 12**	M	9,28	IV	0	0	1	0	0,12	T-LBL	consolidation	LNH-97
**T-LBL 13**	F	12,05	IV	0	0	1	0	na	T-LBL	mantainance	LNH-97
**T-LBL 14**	M	9,07	III	0	0	1	1	na	T-LBL	not achieved	EuroLB02
**T-LBL 15**	M	9,60	IV	0	0	0	0	na	T-LBL	induction	EuroLB02
**T-LBL 16**	M	15,90	III	0	0	0	1	0,13	T-LBL	not achieved	EuroLB02
**T-LBL 17**	M	7,24	III	1	0	0	0	na	T-LBL	induction	EuroLB02
**T-LBL 18**	F	15,95	III	0	0	0	1	na	T-LBL	not achieved	EuroLB02
**T-LBL 19**	M	8,38	IV	0	0	1	0	0,25	T-LBL	consolidation	EuroLB02
**T-LBL 20**	M	14,20	III	0	0	0	0	na	T-LBL	induction	EuroLB02
**T-LBL 21**	M	3,06	III	0	0	1	0	na	T-LBL	induction	EuroLB02
**T-LBL 22**	M	11,17	III	0	1	1	0	na	T-LBL	induction	EuroLB02
**T-LBL 26**	M	3,27	IV	0	0	0	0	0,13	T-LBL	consolidation	EuroLB02
**T-LBL 27**	M	12,16	III	0	0	1	0	na	T-LBL	not achieved	EuroLB02
**T-LBL 28**	M	13,92	III	0	0	1	0	na	T-LBL	induction	EuroLB02
**T-LBL 29**	M	9,45	IV	0	1	1	1	na	T-LBL	consolidation	LNH-97
**T-LBL 30**	F	18,53	IV	1	0	0	0	0,21	T-LBL	re-induction	LNH-97

Table S2. List of *q*-values and local FDR. List of significant differentially activated or expressed proteins between T-LBL patients with poor and good prognosis. Non parametric two-sample Wilcoxon tests (Mann-Whitney tests) were applied to find Multiplicity corrections were applied following two well-known methods: Storey's false discovery rate (qvalue) and Storey's local false discovery rate (local FDR)[22].

Proteins	*q*-values	Local FDR
PRAS40 T246	0.24889765	0.032497619
c-MYC	0.024889765	0.044911494
JAK2 Y1007-1008	0.024889765	0.044911494
BCL-XL	0.036778169	0.078696905
BAX	0.036778169	0.078696905
$P27^{Kip1}$	0.036778169	0.078696905
PKCΔ T505	0.096783894	0.135361967
STAT6 Y641	0.103353231	0.148523881

Table S3. List of primary antibodies selected for RPPA staining. Each antibody was previously validated for single band specificity by Western Blot. Proteins to be analyzed in this study were chosen because belonging to the AKT/mTOR, JAK/STAT, RAS/MAPK, cell cycle and T-cell Receptor signaling pathways.

Antibody	Company	Catalog number	dilution
4EBP1 S65 174A9	Cell Signaling Technology	9456	1:50
Acetyl-CoA Carboxylase 1 S79	Millipore	04-1009	1:1000
AKT S473	Cell Signaling Technology	9271	1:75
AKT T308	Cell Signaling Technology	13038	1:150
AKT TOT	R&D	MAB1775	1:100
ALK Y1604	Cell Signaling Technology	3341	1:100
AMPKα T172	Cell Signaling Technology	2535	1:50
BAX	Cell Signaling Technology	2772	1:50
BCL-XL	Cell Signaling Technology	2762	1:75
BCL-2 S70	Cell Signaling Technology	2827	1:35
B-RAF S445	Cell Signaling Technology	2696	1:50
C-MYC (9E10)	Millipore	OP10	1:100
Cleaved CASPASE 7 (D198)	Cell Signaling Technology	9491	1:100
CDK2 (78B2)	Cell Signaling Technology	2546	1:50
CK2α	Cell Signaling Technology	2656	1:100
CYCLIN B	BD bioscience	610220	1:100
CYCLIN E	BD bioscience	51-1459GR	1:100
eIF4G S1108	Cell Signaling Technology	2441	1:75
ERK1/2 T202/Y204	Cell Signaling Technology	9101	1:100
FAK Y397	BD bioscience	611806	1:75
GSK3α/β S21-9	Cell Signaling Technology	9331	1:500
HSP70/HSP72	Enzo Life Science	ADI-SPA-810	1:75
JAK1 Y2022/2023	Cell Signaling Technology	3331	1:50
JAK2 Y1007-1008	Cell Signaling Technology	3771	1:400
LCK TOT (L22B1)	Cell Signaling Technology	2657	1:300
LCK Y505	Cell Signaling Technology	2751	1:50
LKB1 S428	Cell Signaling Technology	3051	1:100
LKB1 TOT (27D10)	Cell Signaling Technology	3050	1:100
MEK1/2 S217/221 (41G9)	Cell Signaling Technology	9154	1:200
mTOR S2448	NOVUSBIO	NBP1-51413	1:100
mTOR TOT (L27D4)	Cell Signaling Technology	4517	1:100
NOTCH1 TOT (C37C7)	Cell Signaling Technology	3439	1:100
cleaved NOTCH (D3B8)	Cell Signaling Technology	4147	1:75
p21 Waf1/Cip1 (12D1)	Cell Signaling Technology	2947	1:30
p27 Kip1 (57)	BD bioscience	610241	1:50
p38 T180/Y182 (D3F9)	Cell Signaling Technology	4511	1:100
p53	Cell Signaling Technology	9282	1:75
p70 S6K T389	Cell Signaling Technology	9205	1:75
PDGFR receptor β (Y751) (88H8)	Cell Signaling Technology	3166	1:1000
PDK1 S241	Cell Signaling Technology	3061	1:100
PIM-1	Bethyl	A300-313A-T	1:75
PKCα S657	Millipore	06822	1:400
PKCΔ T505	Cell Signaling Technology	9374	1:100
PKCθ T538	Cell Signaling Technology	9377	1:100
PKCζ/λ T410/403	Cell Signaling Technology	9378	1:50
PRAS40 T246	BioSource	44-1100	1:200
PTEN S380	Cell Signaling Technology	9551	1:100

PTEN TOT	Cell Signaling Technology	9552	1:100
RB S780	BD bioscience	558385	1:50
RB TOT	BD bioscience	554136	1:100
S6RP S235/236	Cell Signaling Technology	2211	1:250
SF3b155/SAP155	Bethyl	A300-996A-T	1:200
SRC Y416	Cell Signaling Technology	2101	1:50
SRC Y527	Cell Signaling Technology	2105	1:50
SRSF3	Abcam	ab73891	1:200
SRSF6	Abcam	ab140602	1:100
STAT1 Y701	Cell Signaling Technology	9171	1:50
STAT3 S727	Cell Signaling Technology	9134	1:50
STAT3 Y705	Cell Signaling Technology	9145	1:50
STAT 5 Y694	Cell Signaling Technology	9351	1:30
STAT6 Y641	Millipore	06937	1:75
TYK2 Y1054/1055	Cell Signaling Technology	9321	1:50
USP7	Bethyl	A300-033A-7	1:1000
YAP S127	Abcam	Ab76252	1:50

Table S4. Correlations between JAK1/2-STAT6 RPPA phosphorylation intensity levels and patient clinical and biologic characteristics and clinical outcome. Correlations were calculated based on Fisher test.

		**JAK1/2-STAT6 HIGH**	**JAK1/2-STAT6 LOW**	***p-VALUE***
OUTCOME	POOR	n = 6	n = 1	0.06347
	GOOD	n = 5	n = 10	
NOTCH1 and/or FBXW7 MUTATED	YES	n = 6	n = 6	1
	NO	n = 5	n = 5	
PTEN MUTATED	YES	n= 2	n= 0	0.4762
	NO	n= 9	n= 11	
STAGE	IV	n = 3	n = 5	0.6594
	III	n = 8	n = 6	

Table S5. Combination index (CI) in ALL-SIL, MOLT-4 and RPMI-8402 GC resistant cell lines (MTT test). CIs were calculated at the reported concentration of (A) AS1517499 at scalar dose and dexamethasone at fixed dose of 10 μM for ALL-SIL and 50 μM for MOLT-4. (B) ruxo:dex both at scalar dose with a ratio 10:1 by the Bliss Independence model[24]. Synergy, additivity and antagonism are defined by a CI<1, CI=1 or CI>1, respectively.

A

**AS1517499 μM**	**Combination Index**	
	**ALL-SIL**	**MOLT-4**
0.01	1.97	0.326
0.1	0.989	0.942
1	0.801	0.897

B

**ruxo: dex μM**	**ALL-SIL**	**Combination Index MOLT-4**	**RPMI-8402**
12.5/1.25	0.762	0.640	1.263
25/2.5	0.739	0.465	0.474
50/5	0.833	0.637	0.536
100/10	0.855	0.847	0.529

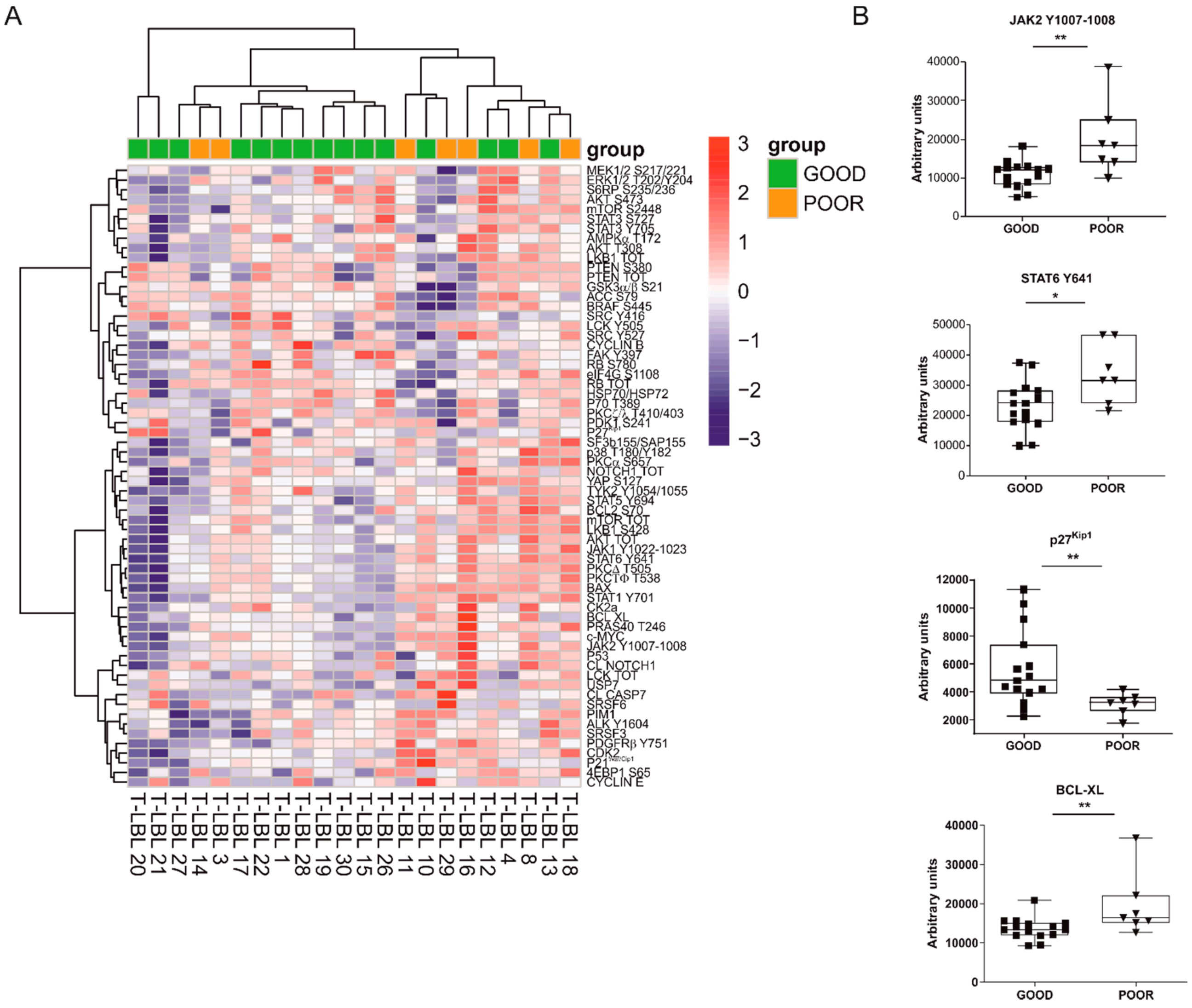

Figure S1. RPPA results. (**A**) Hierarchical cluster analysis with Ward's method of 64 proteins belonging to the most deregulated pathways in cancer analyzed by RPPA in T-LBL pediatric patients with poor prognosis (orange) and good prognosis (green). (**B**) JAK2 Y1007-1008, STAT6 Y641 and BCL-XL measured by RPPA analysis are downregulated in patients with good prognosis (n =15) compared to patients with worse prognosis (n =7), whereas p27^{kip1} is upregulated in good prognosis compared to poor prognosis patients.

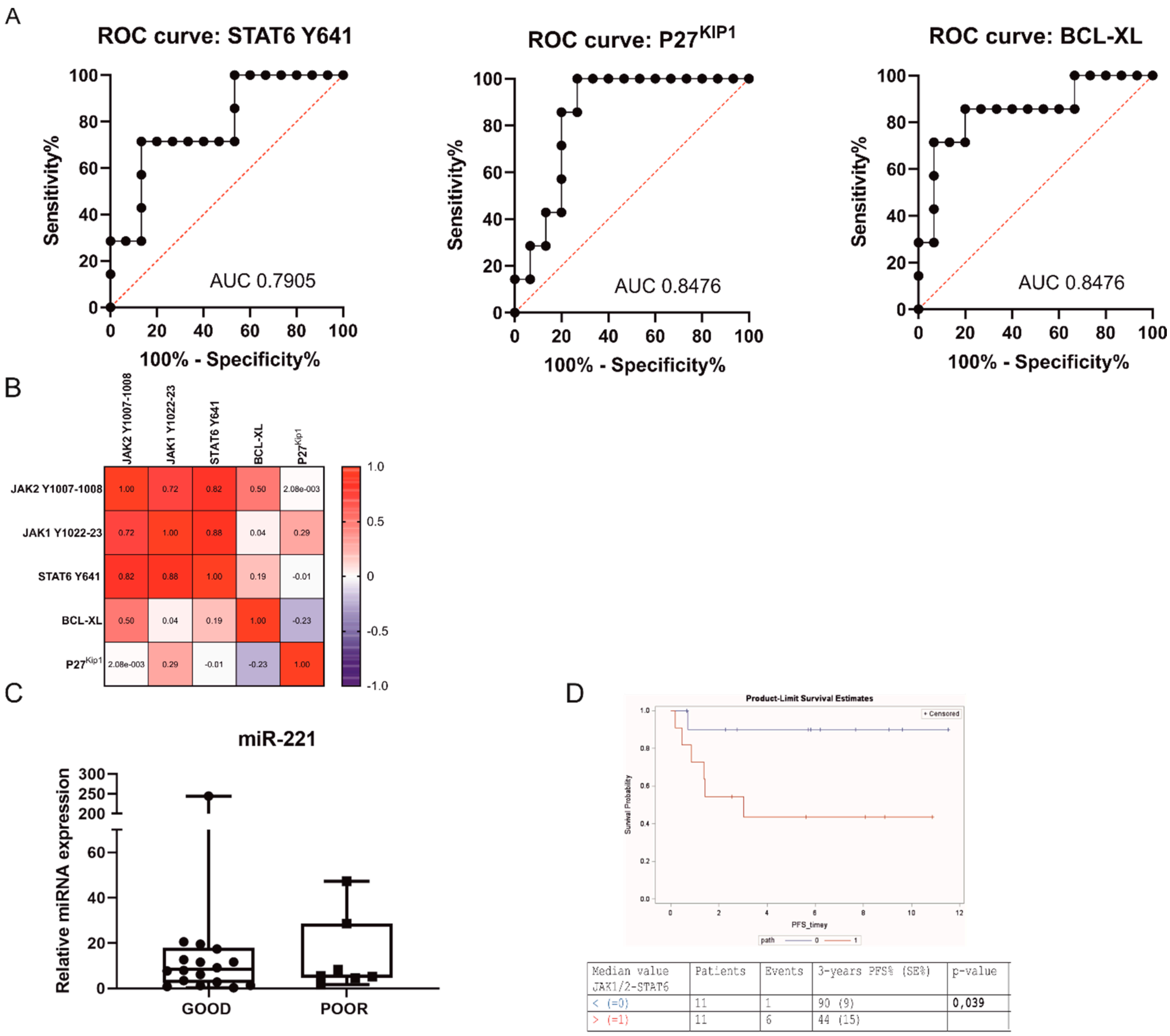

Figure S2. Evaluation of potential biomarkers of prognosis at diagnosis in T-LBL patients and protein correlation matrix (**A**) ROC analysis of the signal intensities obtained by RPPA in T-LBL pediatric patients at diagnosis for STAT6 Y641, BCL-XL and p27^{Kip1}. (**B**) Correlation matrix between the most significant proteins belonging to the JAK1/2-STAT6 pathway (plotted ϱ values). (**C**) Box-and-whisker plots showing the distribution of *miR-221* expression. Median values between T-LBL patients with good and poor prognosis are not significantly different, based on nonparametric test. (**D**) T-LBL patients stratification for the Progression Free Survival (PFS) based on the median JAK1/2-STAT6 activation value (0= below; 1= above).

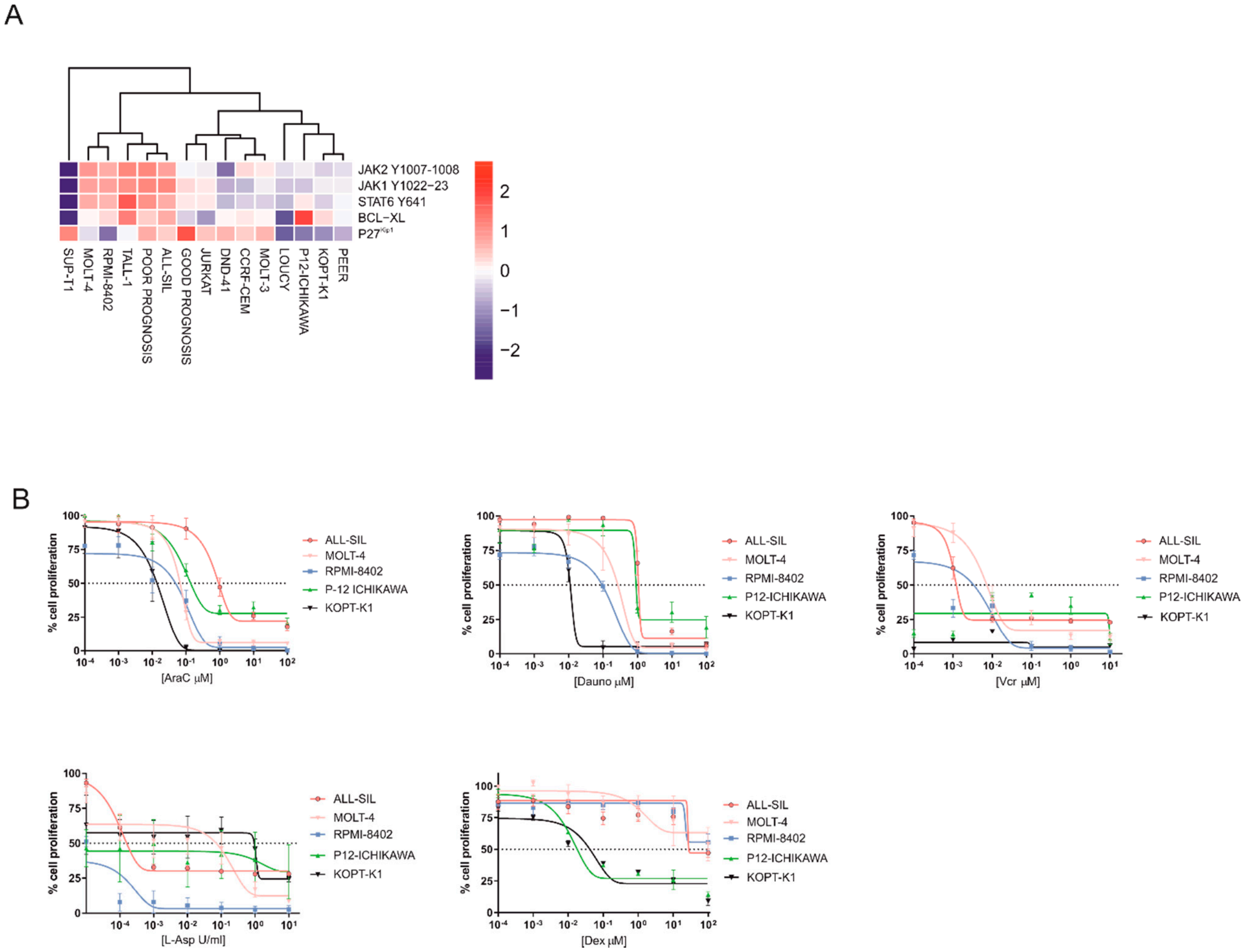

Figure S3. Selection of the *in vitro* model and sensitivity of selected cell lines to the conventional chemotherapeutic compounds. (**A**) Unsupervised hierarchical clustering analysis using the Ward's method and heat map expression levels of proteins belonging to JAK1/2-STAT6 pathway in 13 T-LBL/T-ALL cell lines and in the 2 subgroups of T-LBL patients. Specifically, we plotted the mean RPPA value for good prognosis (n=15) and poor prognosis (n=7) T-LBL patients for each considered protein. (**B**) Cell proliferation of ALL-SIL, MOLT-4, RPMI-8402, KOPT-K1 and P12-ICHIKAWA cells after treatment for 48 hours with dexamethasone, AraC, vincristine, daunorubicin and L-Asparaginase. All experiments were performed at least 3 times, and data are represented as mean ± SEM.

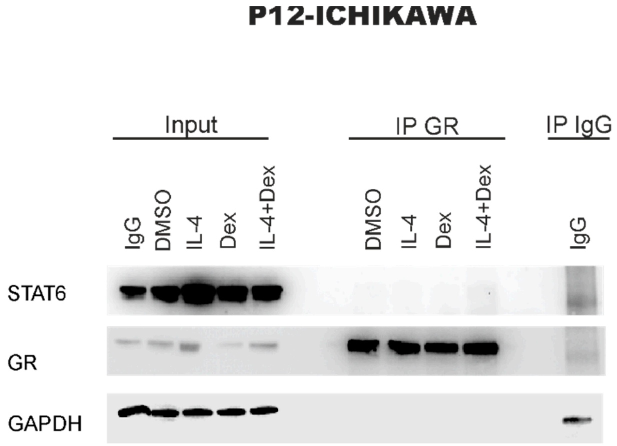

Figure S4. Uncropped images of co-immunoprecipitation of GR and STAT6 protein in P12-ICHIKAWA cells treated with DMSO, IL-4, dexamethasone or the combination of IL-4 and dexamethasone. GAPDH was used as loading control for the input. On the right the IP with the IgG control.

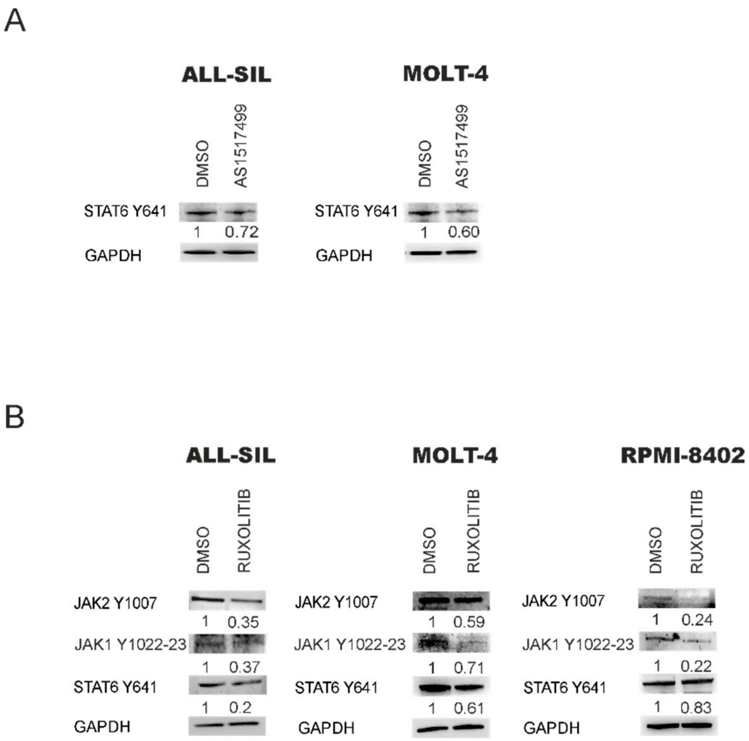

Figure S5. JAK1/2-STAT6 levels after AS1517499 or ruxolitinib treatment in ALL-SIL, MOLT-4 and RPMI-8402. (**A**) WB analysis of the decreased phosphorylation of STAT6 Y641 after 5 minutes of treatment with DMSO or AS1517499 at the concentration corresponding to the GI_{50} in ALL-SIL and MOLT-4 cell lines. (**B**) WB analysis of JAK2 Y1007, JAK1 Y1022-23 and STAT6 Y641 phosphorylation levels in ALL-SIL, MOLT-4 and RPMI-8402. Cells were treated with DMSO only or ruxolitinib for 2 hours at the compound concentrations corresponding to the GI_{50} at 48 hours.

Western Blot uncropped images

Figure 2C, STAT6 Y641 in KOPT-K1 cell line after IL-4 and IL-13 stimulation

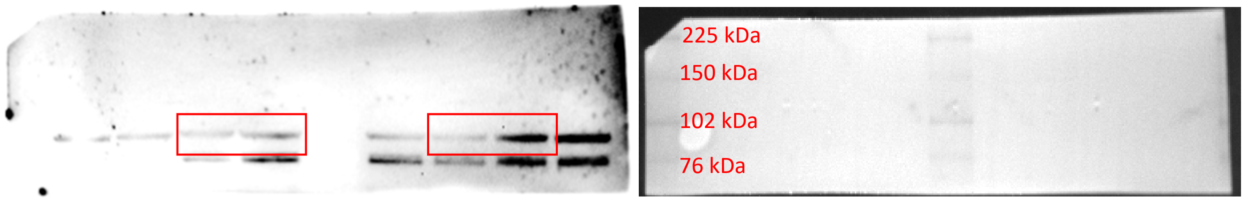

Figure 2C, STAT6 in KOPT-K1 cell line after IL-4 and IL-13 stimulation

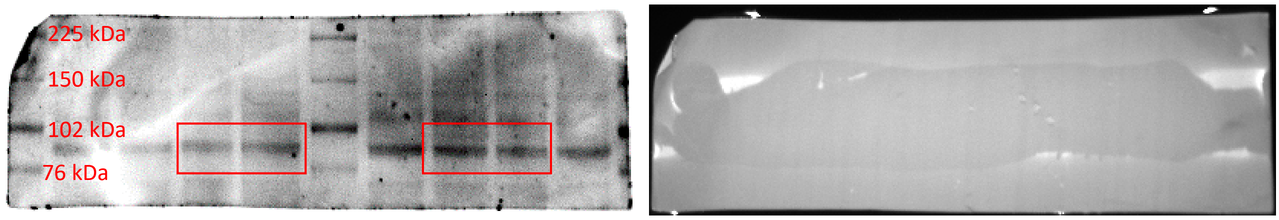

Figure 2C, STAT5 Y694 in KOPT-K1 cell line after IL-4 and IL-13 stimulation

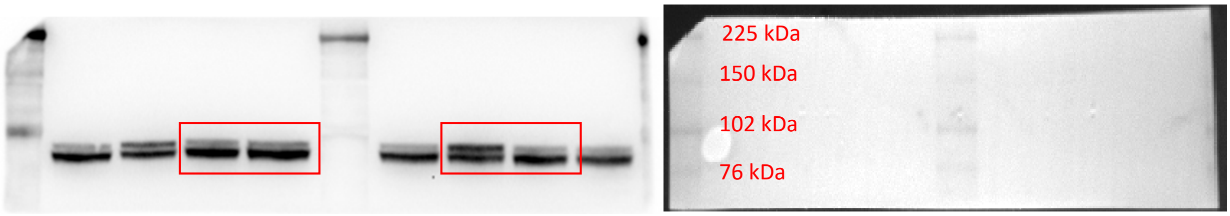

Figure 2C, STAT5 in KOPT-K1 cell line after IL-4 and IL-13 stimulation

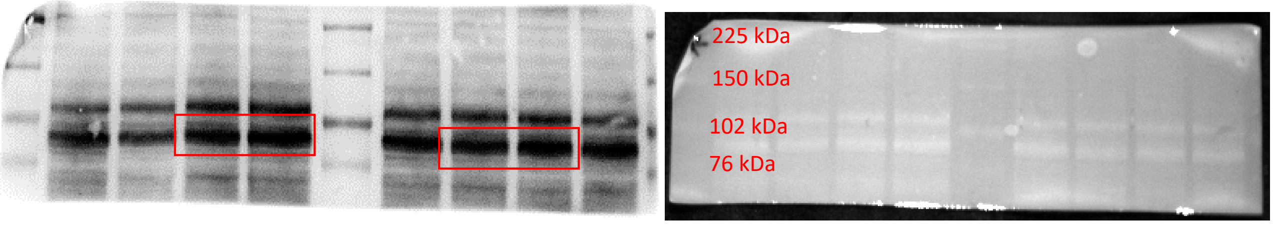

Figure 2C, STAT3 Y705 in KOPT-K1 cell line after IL-4 and IL-13 stimulation

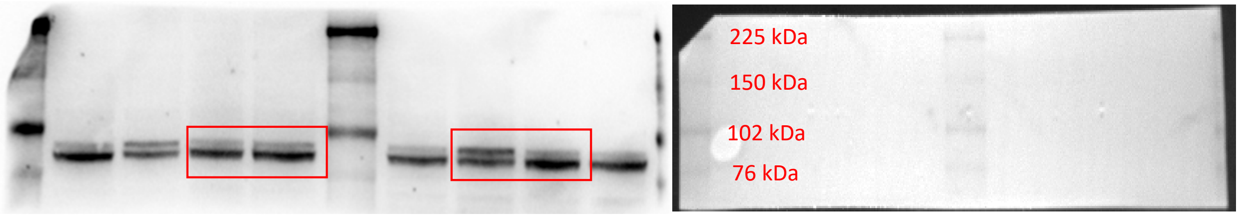

Figure 2C, STAT3 in KOPT-K1 cell line after IL-4 and IL-13 stimulation

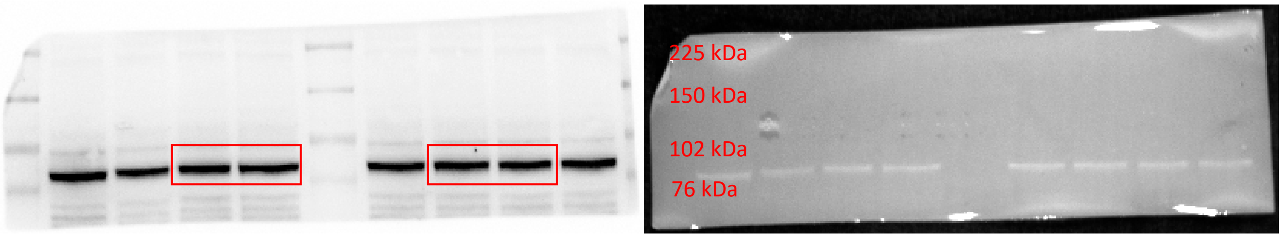

Figure 2C, GAPDH in KOPT-K1 cell line after IL-4 and IL-13 stimulation

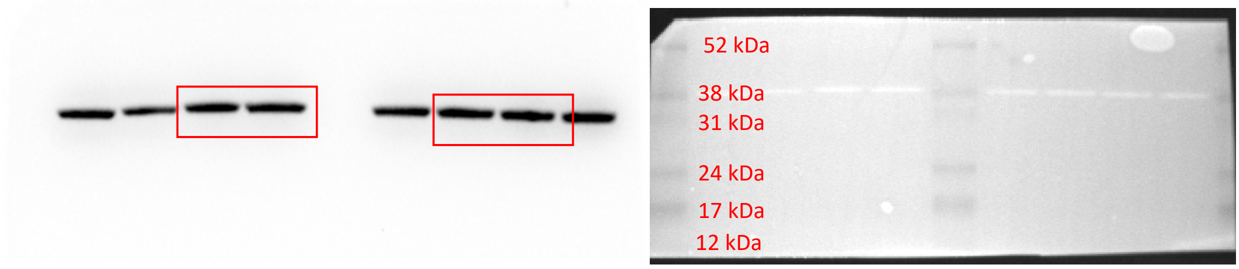

Figure 2C, STAT6 Y641 in P12-ICHIKAWA_cell line after IL-13 stimulation

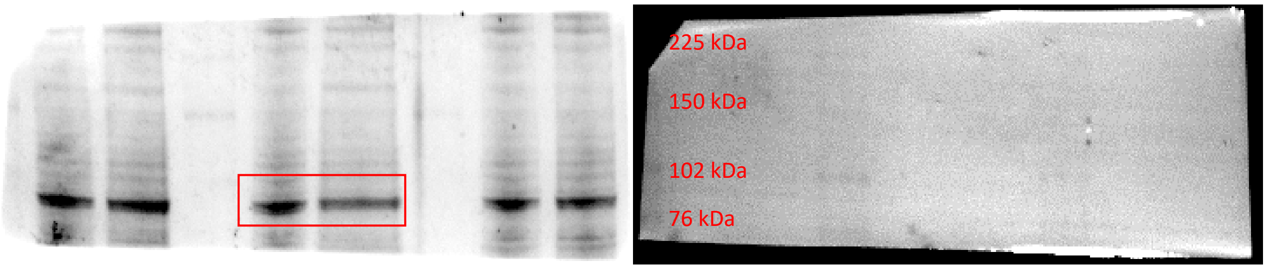

Figure 2C, STAT6 in P12-ICHIKAWA_cell line after IL-13 stimulation

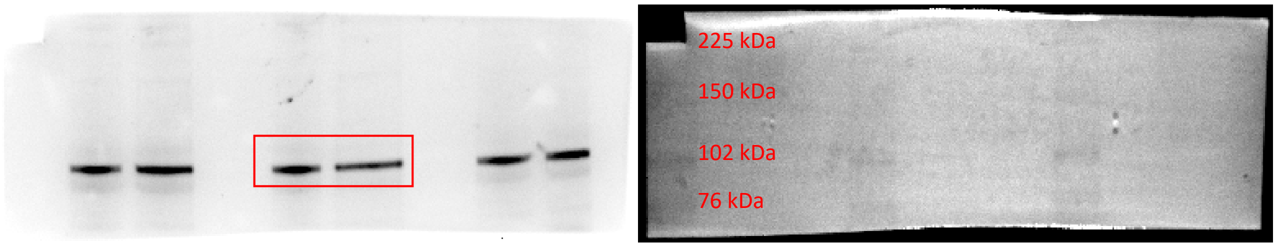

Figure 2C, STAT5 Y694 in P12-ICHIKAWA_cell line after IL-13 stimulation

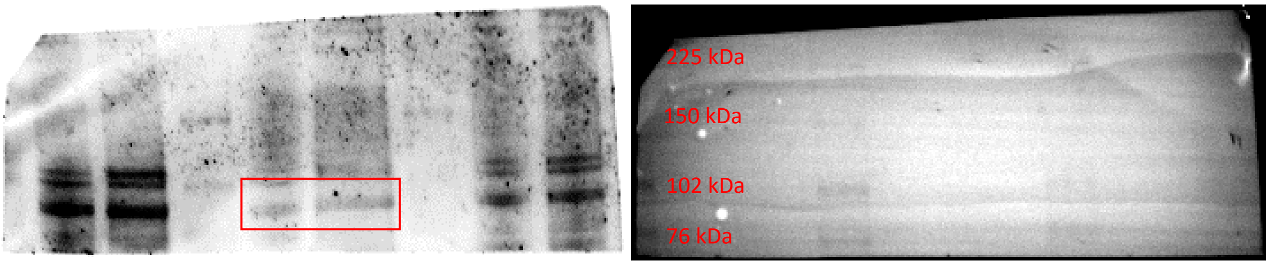

Figure 2C, STAT5 in P12-ICHIKAWA_cell line after IL-13 stimulation

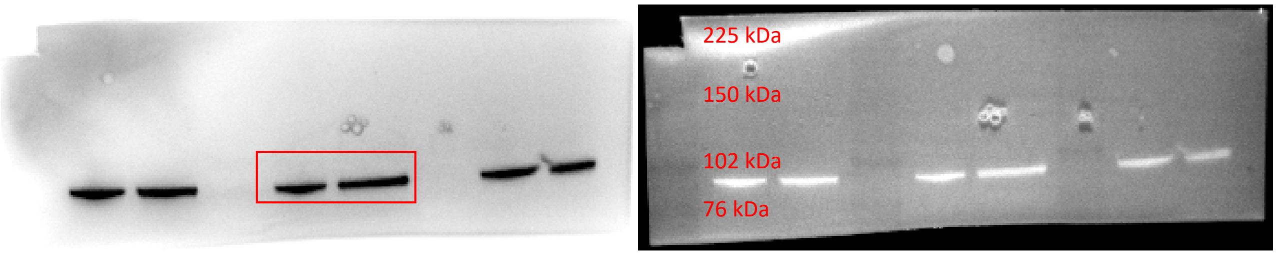

Figure 2C, STAT3 Y705 in P12-ICHIKAWA_cell line after IL-13 stimulation

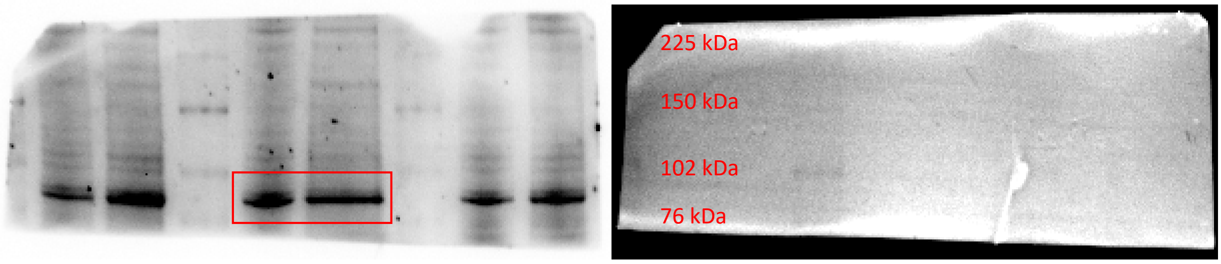

Figure 2C, STAT3 in P12-ICHIKAWA_cell line after IL-13 stimulation

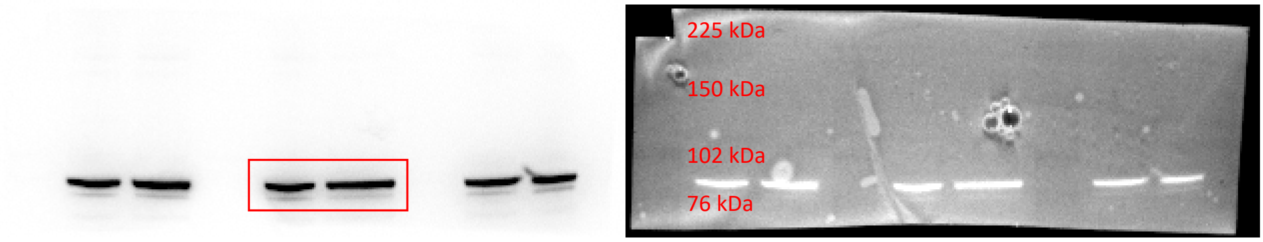

Figure 2C, GAPDH in P12-ICHIKAWA_cell line after IL-13 stimulation

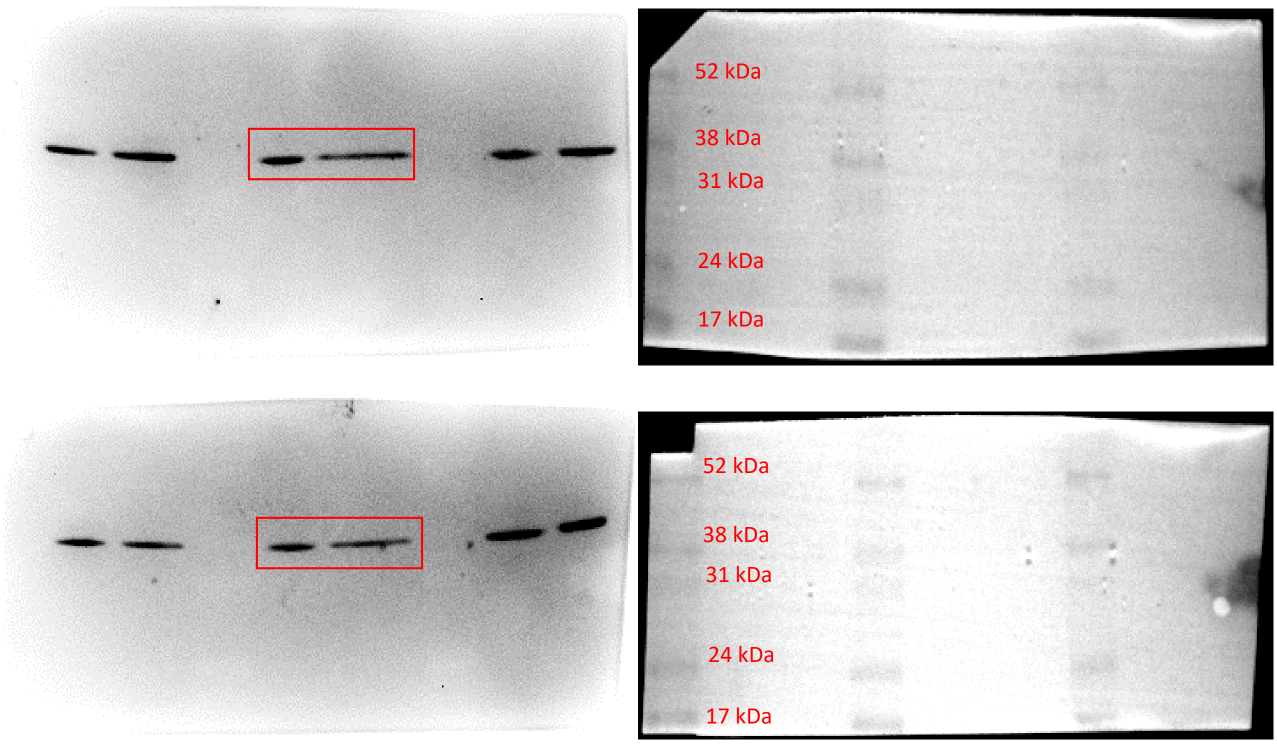

Figure 2C, STAT6 Y641 in P12-ICHIKAWA_cell line after IL-4 stimulation

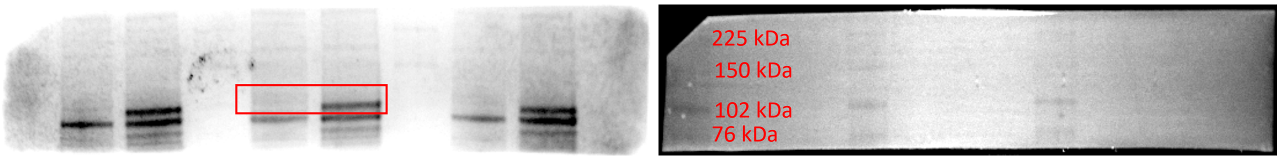

Figure 2C,STAT6 in P12-ICHIKAWA_cell line after IL-4 stimulation

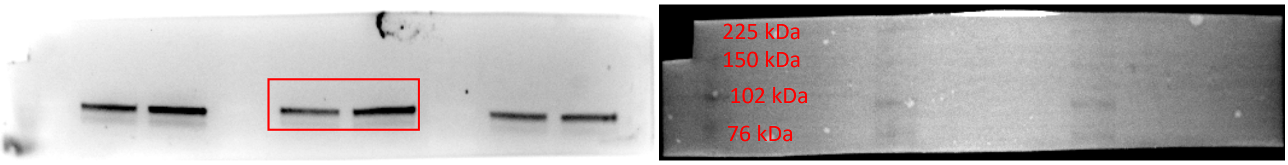

Figure 2C,STAT5 Y694 in P12-ICHIKAWA_cell line after IL-4 stimulation

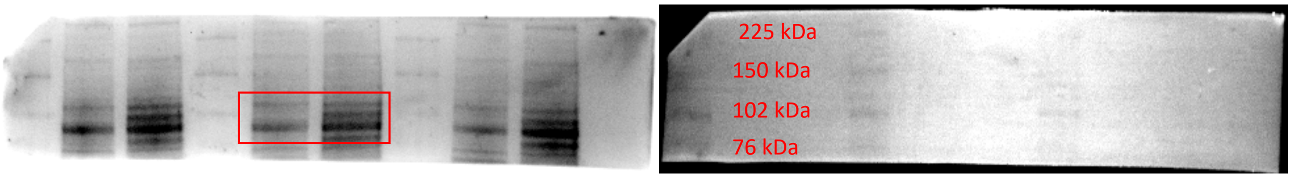

Figure 2C,STAT5 in P12-ICHIKAWA_cell line after IL-4 stimulation

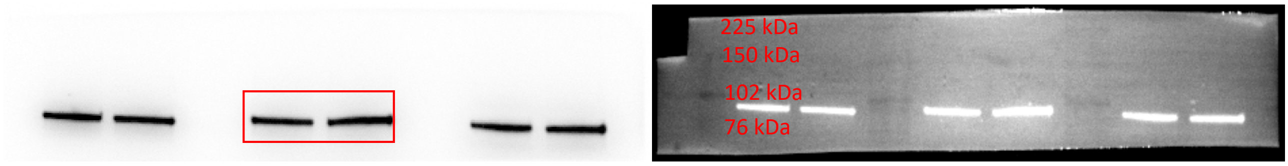

Figure 2C,STAT3 Y705 in P12-ICHIKAWA_cell line after IL-4 stimulation

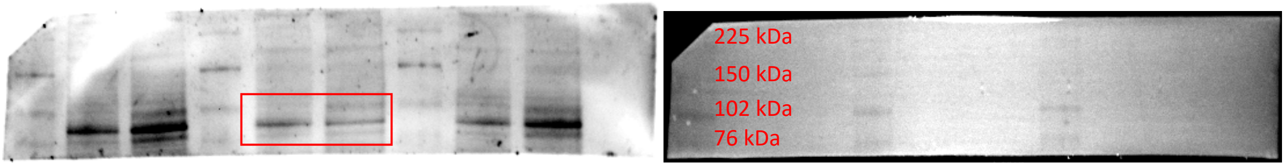

Figure 2C, STAT3 in P12-ICHIKAWA_cell line after IL-4 stimulation

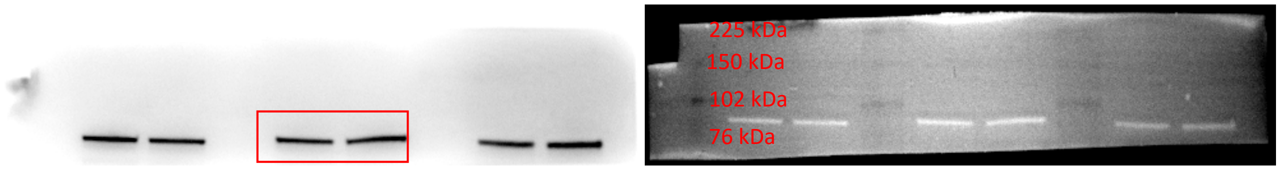

Figure 2C, GAPDH in P12-ICHIKAWA_cell line after IL-4 stimulation

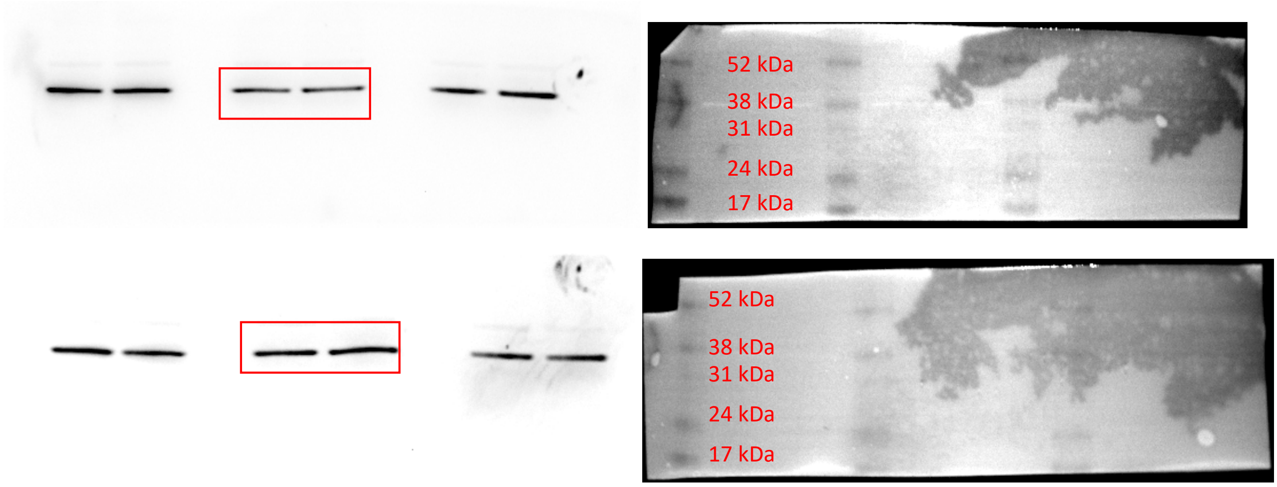

Figure 3A and S4, STAT6 level in after immunoprecipitation in P12-ICHIKAWA cells treated with DMSO, IL-4 alone, dexamethasone alone, or the combination of IL-4 and dexamethasone.

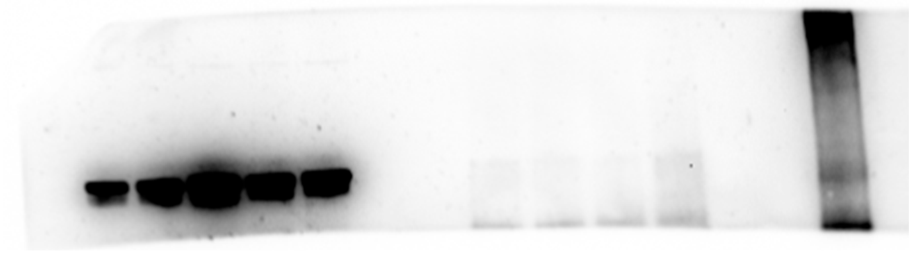

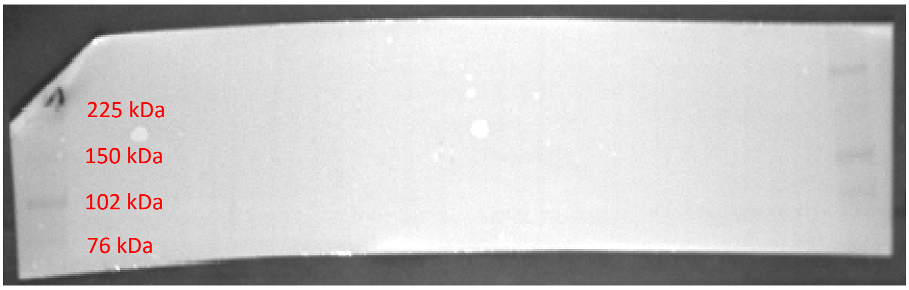

Figure 3A and S4, GR level in P12-ICHIKAWA cell line after immunoprecipitation in P12-ICHIKAWA cells treated with DMSO, IL-4 alone, dexamethasone alone, or the combination of IL-4 and dexamethasone.

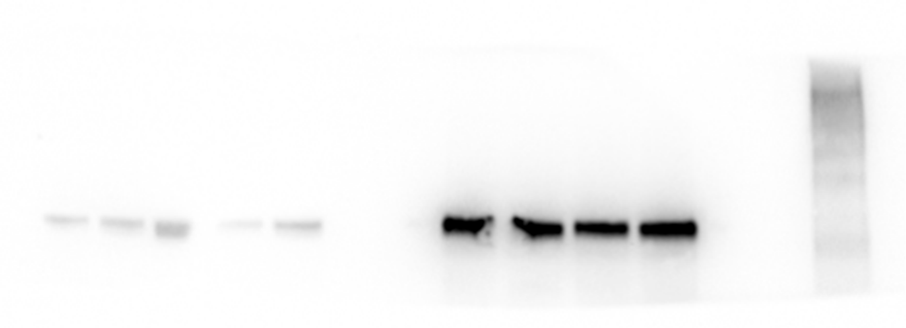

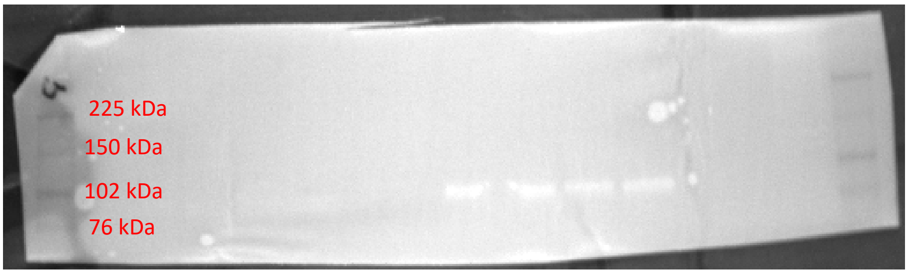

Figure 3A and S4, GAPDH in P12-ICHIKAWA cell line after immunoprecipitation in P12-ICHIKAWA cells treated with DMSO, IL-4 alone, dexamethasone alone, or the combination of IL-4 and dexamethasone.

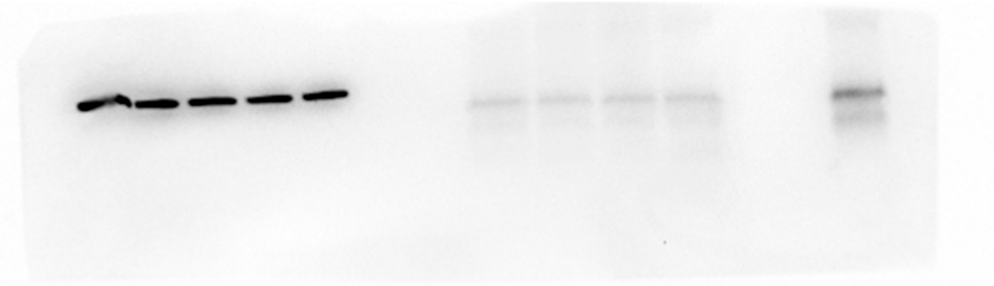

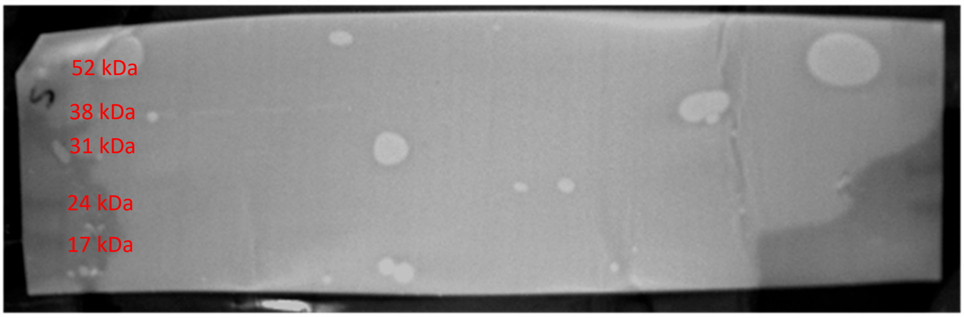

Figure 3C, STAT6 level in P12-ICHIKAWA cell line after STAT6 specific silencing

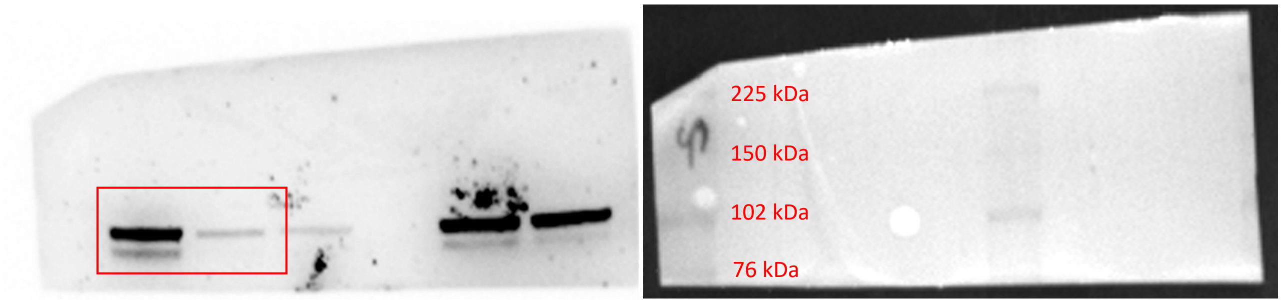

Figure 3C, GAPDH level in P12-ICHIKAWA cell line after STAT6 specific silencing

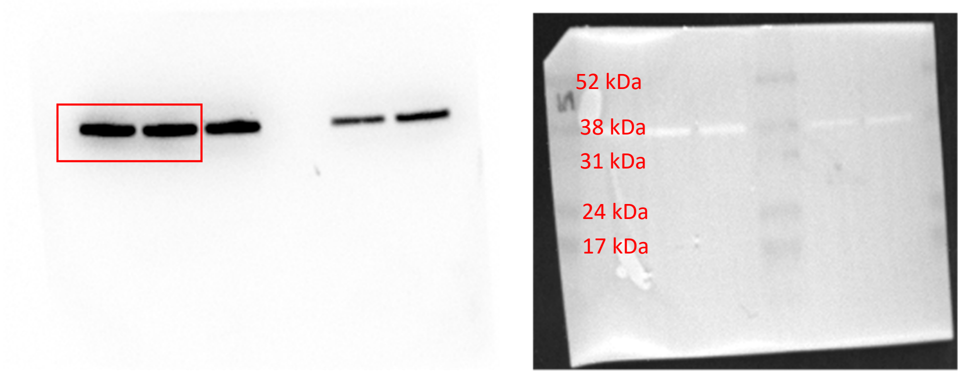

Figure 4A, STAT6 level in ALL-SIL cell line after STAT6 specific silencing

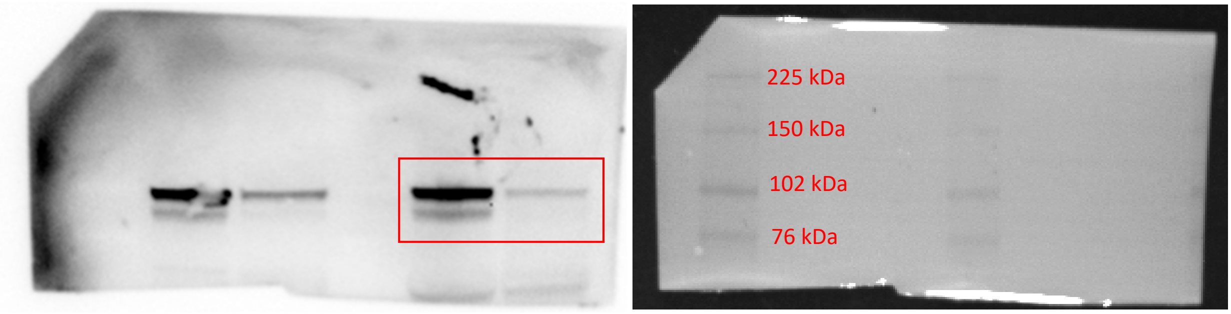

Figure 4A, GAPDH level in ALL-SIL cell line after STAT6 specific silencing

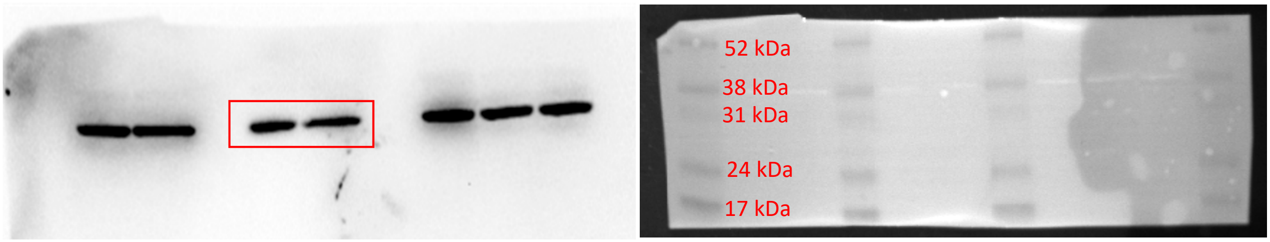

Figure S5, STAT6 Y641 level in ALL-SIL cell line after AS1517499 treatment

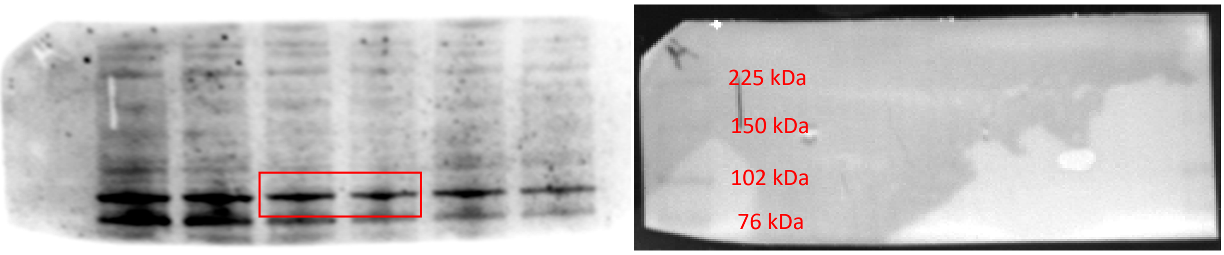

Figure S5, STAT6 Y641 level in MOLT-4 cell line after AS1517499 treatment

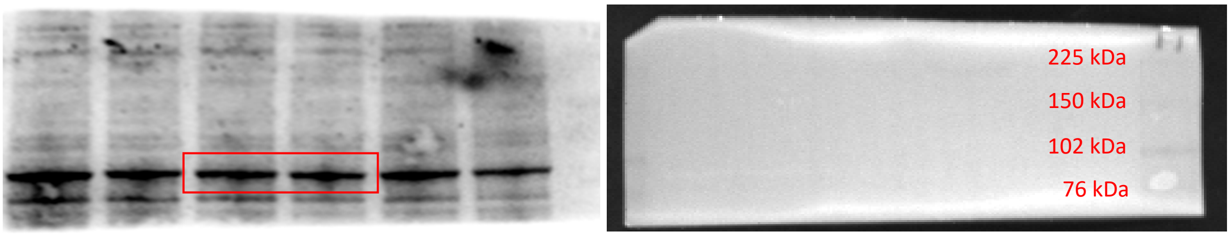

Figure S5, GAPDH level in ALL-SIL and MOLT-4 cell line after AS1517499 treatment

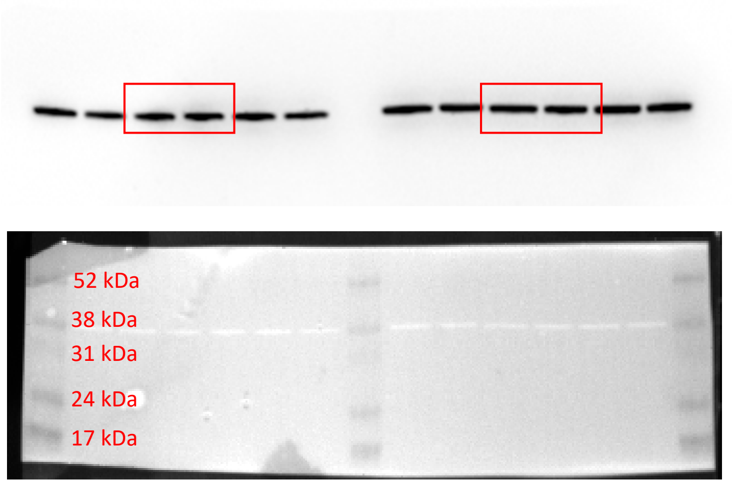

Figure S5, JAK2 Y1007, JAK1 Y1022-23 and STAT6 Y641 level in ALL-SIL cell line after Ruxolitinib treatment

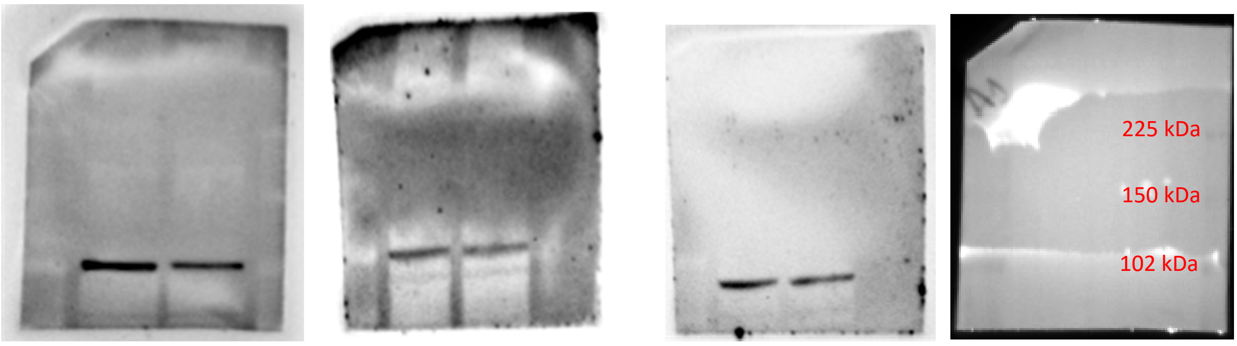

Figure S5, GAPDH level in ALL-SIL cell line after Ruxolitinib treatment

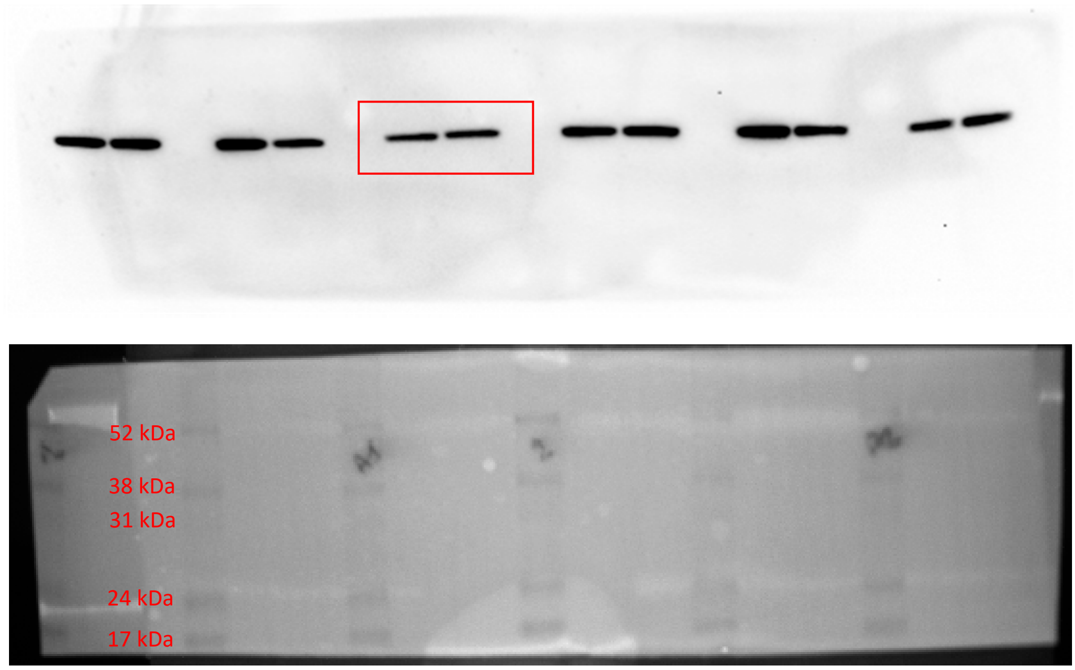

Figure S5, JAK2 Y1007, JAK1 Y1022-23 and STAT6 Y641 level in MOLT-4 cell line after Ruxolitinib treatment

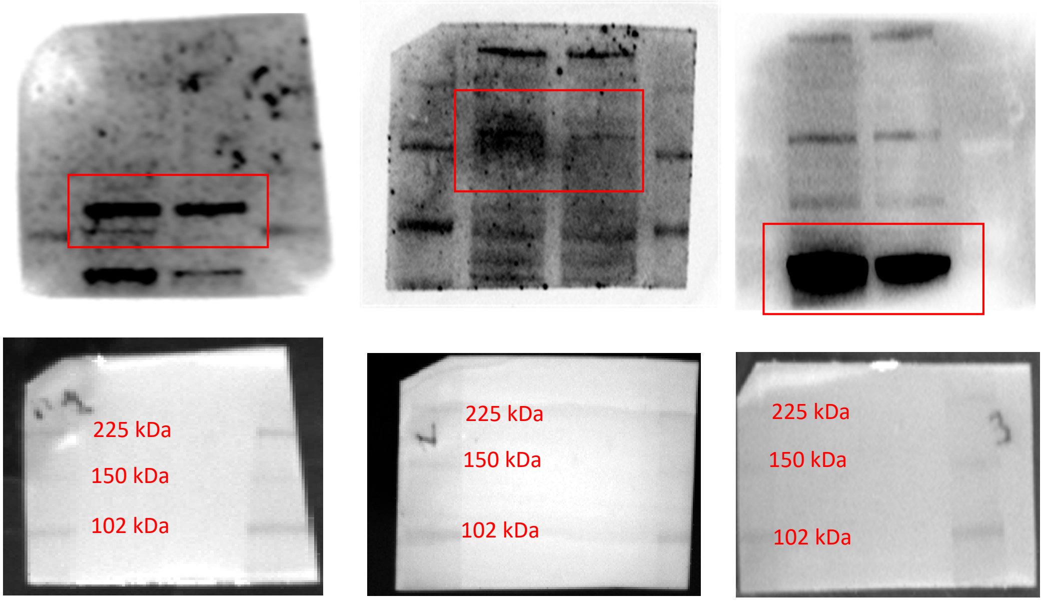

Figure S5, GAPDH level in MOLT-4 cell line after Ruxolitinib treatment

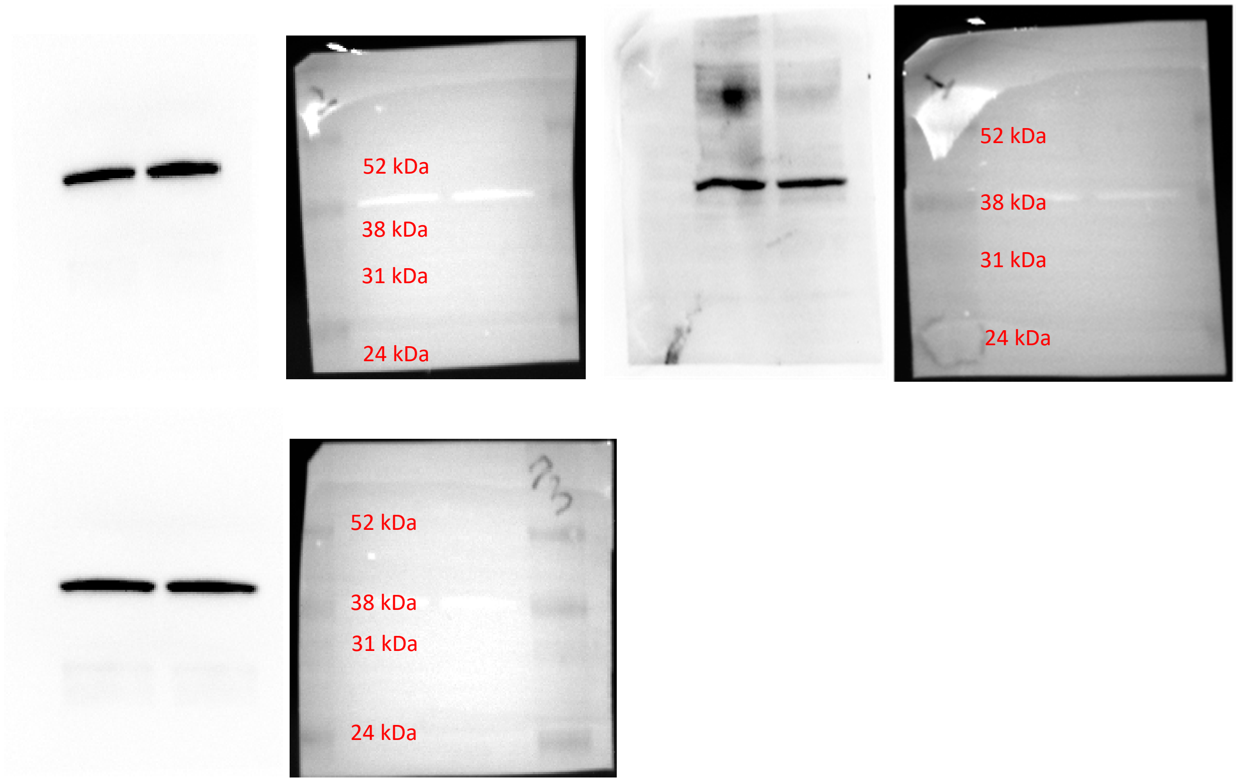

Figure S5, JAK2 Y1007, JAK1 Y1022-23 and STAT6 Y641 level in RPMI-8402 cell line after Ruxolitinib treatment

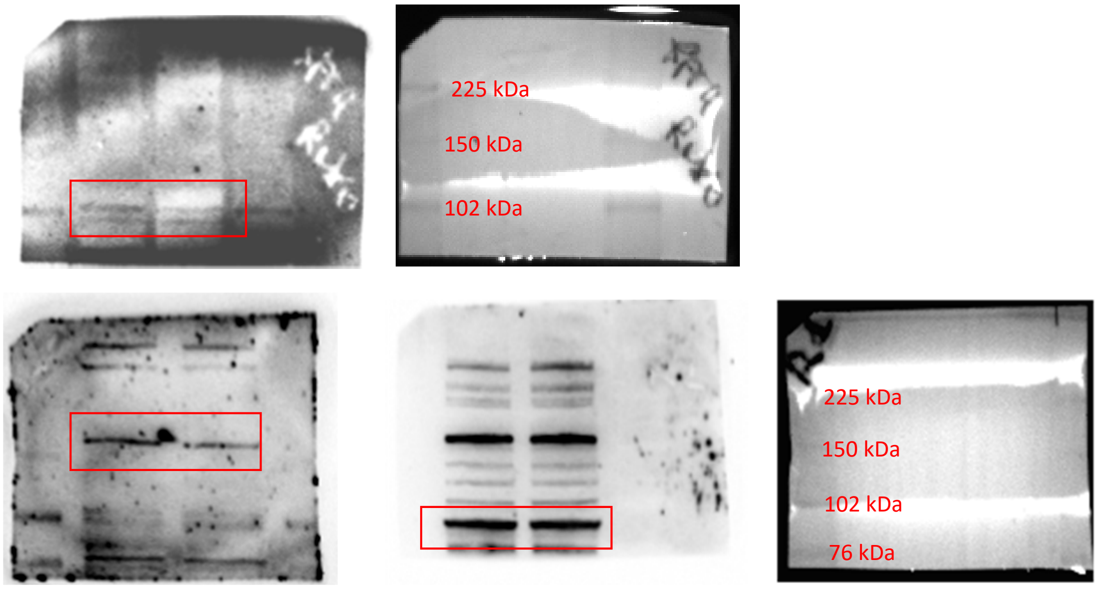

Figure S5, GAPDH level in RPMI-8402 cell line after Ruxolitinib treatment

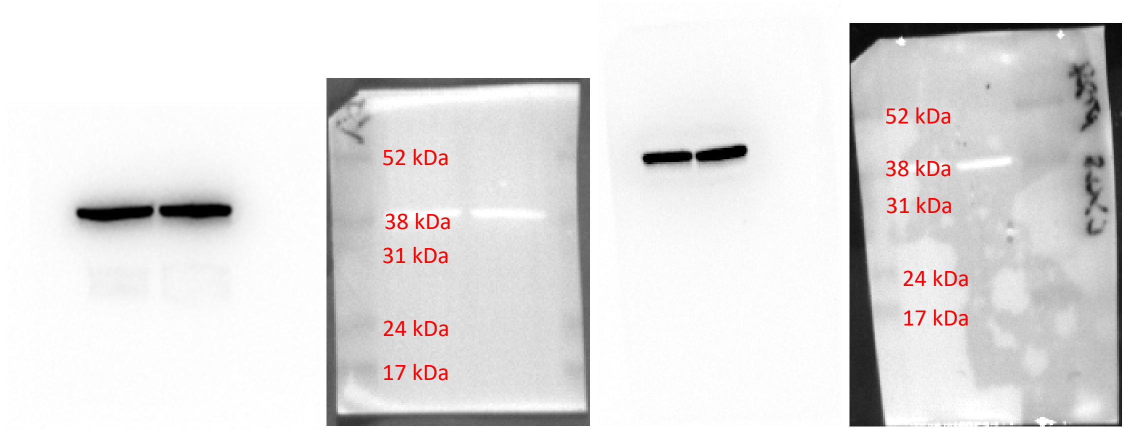

Figure S6. uncropped original western blot.
